# BMP9 knockout impairs pulmonary vessel muscularisation and confers aberrant tamoxifen sensitivity

**DOI:** 10.1007/s10456-025-10017-5

**Published:** 2025-11-12

**Authors:** Benjamin J. Dunmore, Stephen Moore, Rowena J. Jones, Joshua Hodgson, Kathryn Auckland, Mark Southwood, Nichola Figg, Nobuhiro Kikuchi, Martin Bennett, Allan Lawrie, Christopher J. Rhodes, Mark R. Toshner, Stefan Gräf, Wei Li, Nicholas W. Morrell, Paul D. Upton, UK National Cohort Study of Idiopathic and Heritable PAH Consortium, the Uniphy Clinical Trials Network

**Affiliations:** 1 Victor Phillip Dahdaleh Heart and Lung Research Institute, University of Cambridge, Cambridge, CB2 0BB UK; 2 Department of Medicine, School of Clinical Medicine, University of Cambridge, Cambridge, CB2 0QQ UK; 3 MRC Toxicology Unit, Gleeson Building, Tennis Court Road, Cambridge, CB2 1QR UK; 4 National Heart and Lung Institute, Imperial College London, South Kensington Campus, London, SW7 2AZ UK

**Keywords:** Pulmonary arterial hypertension, Pulmonary vascular remodelling, Bone morphogenetic protein, Tamoxifen

## Abstract

Deleterious mutations in the *GDF2* gene, encoding BMP9, are causative of pulmonary arterial hypertension and hereditary haemorrhagic telangiectasia. Paradoxically, BMP9 germ-line knockout (*Gdf2*^−/−^; *Bmp9* KO) and double *Bmp9* KO*/*conditional *Bmp10* cKO (dKO) mice exhibit an attenuated response to PAH-inducing stimuli. We asked whether this contradiction is due to the pathological, physiological, or genetic consequences of BMP9 knockout. In *Bmp9* KO mice we observed reduced pulmonary vascular smooth muscle cell (SMC) coverage and using RNA-seq analysis of *Bmp9* KO mouse lungs identified two novel genes, *COLQ* and *ITGA6*, which were differentially regulated in a human PAH RNA-seq dataset. In order to study BMP10 loss, postnatal tamoxifen treatment was required to induce *Bmp10* cKO. As previously reported, in dKO mice we observed cardiomegaly and splenomegaly, as well as hyperplasia and hemosiderosis in the pulmonary vasculature. Surprisingly, tamoxifen treated *Bmp9* KO control mice phenocopied these pathological changes in dKO mice and downregulated SMC marker gene transcription. Loss of BMP10 is not critical for severe tissue remodelling in the lung, heart, and spleen, rather *Bmp9* KO mice appear sensitive to tamoxifen and BMP9 loss is the primary cause of mild vessel remodelling due to a basal reduction of smooth muscle cell coverage. This study suggests that interaction of the BMP pathway with tamoxifen needs to be carefully considered when studying *Bmp9* KO mice and urges caution in the context of tamoxifen use when studying cardiovascular and pulmonary disease models.

**Supplementary Information:**

The online version contains supplementary material available at 10.1007/s10456-025-10017-5.

## Introduction

The life-limiting disease pulmonary arterial hypertension (PAH) is associated with progressive pathological remodelling of the pulmonary circulation [1]. The pathophysiological mechanism involves obstruction of the small pulmonary arteries due to abnormal smooth muscle cell proliferation impairing blood flow [2]. Consequently, elevation of pulmonary artery pressure causes hypertrophy of the right ventricle, leading to heart failure and premature death [2]. Germ-line mutations in members of the bone morphogenetic protein (BMP) pathway are causative of disease, including *BMPR2* (BMP type-II receptor), *ACVRL1* (encoding activin receptor-like kinase 1, or ALK1), *ENG* (endoglin), the SMAD signalling intermediary *SMAD9* [3–5], and genes encoding ligands BMP9 (*GDF2*) and BMP10 [5, 6]. Interestingly, mutations in *GDF2*, as well as in *ACVRL1*, *ENG* and *SMAD4* are also known to be causal of hereditary haemorrhagic telangiectasia (HHT) [7, 8]. HHT pathology is characterised by telangiectasias, epistaxis and in some cases multiple arteriovenous malformations (AVMs) due to loss of intervening capillaries causing direct interaction between arteries and veins [9]. Pulmonary AVMs affect approximately 50% of HHT patients and result in high-flow right-to-left shunts [10, 11]. Clinical symptoms include shortness of breath and hypoxemia [10]. Patients are at significant risk of stroke, transient ischemic attacks and cerebral abscess as air, thrombi, or bacteria shunt through the AVM, rather than being filtered by the lungs [10]. Thus, the pathologies of PAH and HHT appear distinct.

In endothelial cells, BMP9 and BMP10, which have high sequence homology, activate the canonical SMAD1/5/8 pathway by forming a signalling complex with the BMP type-I receptor, ALK1, and type-2 receptors including BMPR-II; the auxiliary receptor endoglin can further contribute to BMP9 and BMP10 signalling [12, 13]. BMP9 is produced by the liver whereas BMP10 is most highly expressed in the right atrium in adults, and both are considered vascular quiescence factors [14–16]. Promotion of endothelial cell homeostasis by BMP9 signalling includes prevention of endothelial cell apoptosis and permeability [13, 17]. Furthermore, subacute delivery of a BMP9 neutralising antibody in wild type mice induced pulmonary vascular oedema and inflammation [17]. Administration of recombinant BMP9 reversed PAH in three preclinical PAH models [13]. It is worth noting that although BMP9 signals predominantly via ALK1 at physiological concentrations, at higher than physiological levels, it can signal via low affinity type-I receptors such as ALK2 and promote unwanted inflammatory effects [18].

Paradoxically, two studies suggested that loss of BMP9 protects against PAH development in experimental rodent models [19, 20]. Although global *Gdf2*^*−/−*^ loss (*Bmp9* KO) has no impact on mice viability or fertility, defects in lymphatic development have been observed with mesenteric valve maturation and draining efficiency particularly affected [21]. In the first of these studies, knockout of BMP9 and treatment with a BMP9 neutralising antibody suppressed chronic hypoxia-induced pulmonary hypertension (PH) in mice [19]. In comparison to BMP9 loss, BMP10 knockouts die in utero due to failed cardiac trabeculation [22]. To examine phenotypic changes due to BMP10 loss, tamoxifen-inducible knockouts have been generated [20]. Interestingly, conditional knockout of BMP10 does not prevent chronic hypoxia-induced PH [20], and double knockout of BMP9 and BMP10 does not exacerbate the haemodynamic and vessel remodelling observed in the BMP9 knockout models [19, 20].

Taken together there is a clear contradiction regarding BMP9 loss in human genetic findings and experimental mouse models of PH. In this study we set out to understand the mechanisms underpinning the role of BMP9 and BMP10 in pulmonary vascular remodelling. Careful examination revealed that smooth muscle cell coverage was reduced in *Bmp9* KO and double BMP9 and BMP10 knockout (dKO) mice. RNA-seq analysis of *Bmp9* KO mouse lungs identified genes associated with BMP9 loss, but no obvious changes in genes associated with smooth muscle cells. As seen previously, double knockout of BMP9 and BMP10 caused organ hypertrophy and hemosiderosis, surprisingly we also observed this in BMP9 knockout mice treated with tamoxifen which was not considered in previous reports. Collectively, our data suggest that BMP9 deficiency contributes to a remodelled vasculature that is susceptible to tamoxifen treatment. Thus, caution needs to be taken when interpreting studies that aim to determine the compensatory nature of BMP9 and BMP10 in vascular development.

## Materials and methods

### Animals

All procedures, approved by the University of Cambridge Animal Welfare Ethical Review Board, were conducted in accordance with the United Kingdom Animal (Scientific Procedures) Act 1986 and directive 2010/63/EU on the protection of animals used for scientific purposes under the authority of the Project Licences (70/8850—Preclinical therapies for pulmonary hypertension and PP7550697—Mechanisms and treatment of pulmonary vascular diseases). All animal facilities were approved by the United Kingdom Home Office Licensing Authority and conformed to Directive 2010/63/EU of the European Parliament on the protection of animals used for scientific purposes. Mice were housed in a pathogen-free barrier facility under a 12-h light and 12-h dark cycle, and temperature-controlled environment with standard diet and water ad libitum. For all studies, male mice were used.

BMP9 knockout mice (*Gdf2*^−/−^; *Bmp9* KO) were a kind gift from Professor Se-Jin Lee (Johns Hopkins University, MD, USA). Mice were bred on a C57/Bl6 background and crossed through multiple generations, with introduction of fresh C57/Bl6 wild type mice at regular intervals to reduce potential effects from inbreeding. For studies, *Gdf2*^±^ males and females were crossed to generate littermates with the desired genotypes.

A *Bmp10* conditional knock-out (*Bmp10* cKO) mouse was developed by GenOway (Lyon, France). The mouse was developed using cryopreserved material developed by the European Conditional Mouse Mutagenesis Program (EUCOMM). The targeting strategy permitted conditional deletion of exon 2 encoding the C-terminal part of the preproprotein containing the mature form of the BMP10 protein. Insertion of the neomycin cassette and distal loxP site resulted in 65bp and 9bp deletions in intron 1 and downstream region respectively. The resulting line was then revitalised with in vivo Flp-mediated excision and the resulting animals tested by PCR and Southern blot assay. The crossing strategy generated homozygous *Bmp10* cKO mice heterozygous for Gt(ROSA)26Sor^tm1(cre/ERT2)Tyj^ expression (The Jackson Laboratory, Bar Harbor, ME USA). Littermates harbouring *Bmp10*-cKO/Gt(ROSA)26Sor^tm1(cre/ERT2)Tyj^ or *Bmp10* cKO were treated with 40 mg/kg tamoxifen (Sigma-Aldrich, Gillingham, Kent, UK) by intraperitoneal injection once a day for five days, with a two-day recovery period followed by a further 5 days to induce Cre recombination in the *Bmp10* cKO/Gt(ROSA)26Sor^tm1(cre/ERT2)Tyj^ mice. Control mice harbouring the *Bmp10* cKO were dosed with an equivalent volume of corn oil to act as control. Mice were bled from a superficial vessel prior to administration of tamoxifen, one week after the final tamoxifen dose and at the time of phenotyping to harvest serum for BMP9 measurement. Right atria were harvested for in vitro culture, as described below, at the time of phenotyping to assess BMP10 knockdown.

Double knockout mice were generated by crossing *Bmp9 KO* mice with *Bmp10* cKO/Gt(ROSA)26Sor^tm1(cre/ERT2)Tyj^ mice and selectively crossing the offspring to achieve *Bmp9 KO*/*Bmp10* cKO/Gt(ROSA)26Sor^tm1(cre/ERT2)Tyj^ mice and *Bmp9 KO/Bmp10* cKO littermates. For all studies, mouse genotypes from ear biopsies were determined using real time PCR with specific probes designed for each gene (Transnetyx, Cordova, TN).

### Cardiopulmonary phenotyping of mice

All mice were cardiopulmonary phenotyped using closed-chest cardiac catheterisation performed using an MPVS Ultra Single Segment Pressure–Volume Unit in combination with a PVR-1045 pressure–volume catheter (both Millar Instruments, Houston, TX, USA) as previously described. Pressure and volume measurements were acquired and analysed using LabChart Pro software (AD Instruments, Sydney, Australia). For phenotyping studies male mice aged approximately 6 weeks at the start of the study were used, unless otherwise indicated.

### Administration of BMP9 monoclonal antibody

Wild type C57/Bl6 mice were administered weekly for 3-weeks with 5 mg/kg BMP9 antibody (R&D Systems, Abingdon, Oxon, UK) or 5 mg/kg mouse IgG2B isotype control (R&D Systems). For *Bmp10* cKO mice, one week post tamoxifen dosing mice were administered with 5 mg/kg BMP9 antibody or mouse IgG2B isotype control weekly for 3-weeks. One week following the final antibody administration closed-chest cardiac catheterisation was performed as previously described. All antibody studies used male mice aged 4–4.5 weeks at the start of the study.

### BMP9 knockout reversal study by BMP9 treatment

*Bmp9* KO mice aged 4.5–5.5 weeks were dosed by intraperitoneal injection daily for 28 days with either 0.03mg/kg BMP9 (Recombinant Human BMP-9 Protein CF; R&D Systems) in PBS/0.1% mouse serum albumin (MSA; Sigma-Aldrich). A wild type litter mate control group were dosed with an equivalent volume of vehicle (PBS/0.1% MSA) as a control. Mice were bled prior to and at the end of the dosing period from a superficial vessel for confirmation of dose administration. Approximately 4 h following the final dose, mice were culled with an overdose of anaesthetic and tissue taken for further analysis.

### Tissue harvesting and right atrial culture

After catheterisation and exsanguination, mice were dissected. The chest was opened, and the trachea exposed. The right lung lobes were tied off with suture to prevent inflation. An incision was made and a 22-gauge Venflon™ cannula (BD Biosciences, Wokingham, Berks, UK) inserted. The left lung lobe was then inflated by gravity with 10% neutral-buffered formalin starting at a 3ml volume in a 5ml syringe, with the base of the syringe at 6 cm above the prone mouse (approximately 150 µl perfused into the lung lobe). The lungs were inflated and fixed for 10 min. During this time, the liver was dissected, and the spleen and femur were excised. At the end of the 10-min lung inflation period, the left lobe was tied off with suture. The right atrium was removed and treated as below. The other tissues were then harvested and weighed accordingly. Each fresh right atrium was washed in PBS and then placed in 200 µl culture media (DMEM containing 10 mM HEPES, 1 mM sodium pyruvate, 4.5 mM glucose, 20 mM creatine phosphate and antibiotic/antimycotic) in a 2 ml microcentrifuge tube. Four small holes were made in the lid of the tube with a 23-gauge needle and the tube placed at 37 °C in a humidified 5% CO_2_ incubator for 24 h. The atrium was then removed, blotted, and weighed prior to being snap frozen. The conditioned medium was centrifuged at 1500 × g for 10 min and aliquots prepared and frozen.

### BMP9 ELISA

The BMP9 ELISA detects the free BMP9 growth factor domain (GFD) and Pro:BMP9, but not *ProBMP9* [23]. For serum BMP9 measurements, high binding 96‐well ELISA plates (Greiner Bio-One, Stonehouse, Gloucs, UK) were coated with 0.5 µg/well anti‐human BMP9 (MAB3209, R&D Systems) in PBS (Thermo Fisher Scientific, Maidenhead, Berks, UK) and incubated overnight at 4°C in a humidified chamber. Plates were washed with PBS containing 0.05% Tween 20 (PBS‐T), followed by blocking with 1% (w/v) bovine serum albumin (BSA; Thermo Fisher Scientific) in PBS (PBS/1% BSA) for 2 h at RT. Serum samples (25 μl) were premixed with 25 µl PBS/1% BSA plus 50 μl 2X assay diluent (PBS/1% BSA containing 1% Triton X‐100, 0.4% goat serum (GS) and 9 mM EDTA) to a total volume of 100 μl. Recombinant human BMP9 standards (4.88–5000 pg/ml) were prepared in the same final concentrations of additives. After washing, biotinylated anti‐human BMP9 detection antibody (0.04 µg/well BAF3209; R&D Systems) was added in PBS/1% BSA containing 0.2% GS. After further washing, Streptavidin-HRP (R&D Systems) was diluted in PBS/1% BSA according to the lot dilution stated by the manufacturer and then added. The ELISA was developed with Substrate Reagent (R&D Systems) and absorbance measured at 450 nm corrected to the absorbance at 570 nm. Unknown values were extrapolated from the standard curve using a four‐parameter log curve fit. All values are presented as the concentration of the BMP9‐GFD. All samples were measured in duplicate where serum volumes permitted.

### BMP10 GFD ELISA

For measurement of BMP10 in conditioned right atrial medium, a commercial BMP10 ELISA kit was used (DY2926; R&D Systems) plates were coated with 0.2 µg/well of anti‐human BMP10 (MAB2926; R&D Systems) and blocked as above. Recombinant growth factor domain and prodomain BMP10 variants were manufactured as previously described [24]. Right atrial conditioned media samples (10 µl) were premixed with 40 μl PBS/1% BSA plus 50 μl 2X assay diluent (PBS/1% BSA containing 1% Triton X‐100, 0.4% goat serum (GS) and 9 mM EDTA) to a total volume of 100 μl. Recombinant human BMP10 standards (4.88–5000 pg/ml) were prepared in the same final concentrations of additives. After washing, biotinylated anti‐human BMP10 detection antibody (0.1 µg/well; R&D Systems) was added in PBS/1% BSA containing 0.2% GS. After further washing, Streptavidin-HRP (R&D Systems) was diluted in PBS/1% BSA according to the lot dilution stated by the manufacturer and then added. The ELISA was developed with Substrate Reagent (R&D Systems) and absorbance measured at 450 nm corrected to the absorbance at 570 nm. Unknown values were extrapolated from the standard curve using a four‐parameter log curve fit. All samples were measured in duplicate.

### Immunohistochemistry

Pulmonary arteriolar muscularization was assessed on paraffin-embedded sections of fixed mouse lung tissue (3.5 μm thick) labelled with monoclonal mouse anti–smooth muscle α-actin (αSMA) (clone 1A4) or polyclonal rabbit anti-von Willebrand Factor (vWF) (both Dako, Ely, Cambs, UK) antibody. To detect αSMA staining, either the Animal Research Kit, peroxidase (Dako) or Mouse on Mouse Elite Immunodetection kit, peroxidase (Vector Laboratories, Newark, CA, USA) was used in accordance with the manufacturer’s instructions. To detect vWF staining, a Vectastain Elite ABC-HRP kit, peroxidase was used (rabbit IgG; Vector Laboratories). Antibody staining was visualized using 3–3 diaminobenzidine hydrochloride as substrate-chromogen and counterstained with Carrazzi’s haematoxylin (Dako). Pulmonary arteriolar muscularization was assessed by identifying alveolar ducts and categorizing the accompanying intra-acinar artery as non-muscularized, partially muscularized, or fully muscularized by the degree of αSMA immunostaining. A minimum of 20 vessels with diameters ranging from 25 to 75 μm were categorized per animal. Wall thickness was evaluated by identifying small arteries (< 100 μm) proximal to the terminal epithelial bronchioles. The diameter and thickness of the artery were measured after immunostaining for SMA. To measure wall thickness, measurements were taken in four different positions of the artery with a minimum of 10 arteries assessed in each lung section. Perl’s (Prussian Blue) iron stain was conducted using the Richard-Allan scientific iron stain (Epredia, Runcorn, Ches, UK) or Iron Stain Kit (Abcam, Cambridge, Cambs, UK), as per the manufacturer’s instructions. Where indicated haematoxylin–eosin (H&E) staining was conducted. Slides were labelled with a unique anonymous identifier, and the operator was blinded to the genotypes and or/treatments for each mouse.

### Whole slide scanning and image analysis

All slides were scanned on a Hamamatsu Nanozoomer XR (Welwyn Garden City, Herts, UK) and images saved in.NPDI image format and quantified using Visiopharm 2023.09 (Hørsholm, Denmark). In brief, using the H&E-stained slide, differences in pixel colour intensity were used to distinguish between lung parenchyma and alveolar air spaces, with areas measured in µm^2^ and then normalised. Using the Perl’s (Prussian Blue) image, a Visiopharm brightfield nuclei detection APP was used identify all nuclei and then 'blue' nuclei reclassified by pixel colour intensity. The numbers of total and Perl’s-positive cells were then measured per section.

### Immunoblotting

Frozen lung tissues were homogenized in lysis buffer (250 mM Tris–HCl [pH 6.8], 4% SDS, 20% vol/vol glycerol, and 1 × EDTA-free protease inhibitor cocktail; Roche) and sonicated before centrifugation for 15 min at 15,000×*g*. Using the Bio-Rad Lowry assay (Bio-Rad Laboratories), protein concentrations were determined against a BSA standard. Cell lysates (20 μg protein) were separated by SDS-PAGE. Using semidry blotting, proteins were transferred to polyvinylidene fluoride membranes (GE Healthcare). The membranes were blocked and probed with a monoclonal mouse anti–smooth muscle α-actin (αSMA) (clone 1A4; Dako) or anti-Desmin (clone D33; Dako). After washing, the blots were incubated with a secondary anti-mouse horseradish peroxidase antibody (Dako) for 1 h at room temperature. Blots were reprobed with a monoclonal antibody for α-tubulin (clone DM1A; Sigma-Aldrich) or β-actin (clone AC-74; Sigma-Aldrich) as a loading control. Enhanced chemiluminescence was used to detect proteins (GE Healthcare) and ImageJ software was used to assess densitometry.

### RNA extraction from tissues

Mice were killed with an overdose of pentobarbital and exsanguinated prior to removal of lung tissue, which was immediately frozen in liquid nitrogen for RNA isolation. RNA was extracted from cryopreserved lungs in TRIzol™ (Invitrogen, Paisley, Renfrewshire, UK) with homogenization of each sample using an autoclaved stainless-steel bead (Qiagen, Manchester, Greater Manchester, UK) in a tube, with tubes homogenised on a TissueLyser II (Qiagen) in pre-chilled racks (4°C) at 25 Hz for 5 min. Chloroform (Sigma-Aldrich) was added to the homogenised tissue and shaken for 30 s to form an emulsion. The TRIzol/chloroform emulsion was added to 5PRIME phase-lock heavy tubes (Thermo Fisher Scientific) and centrifuged to separate the upper aqueous layer. The aqueous layer was combined with an equal volume of cold (-20°C) iso-propanol (Sigma-Aldrich) and incubated at -70 °C for 30 min to precipitate the RNA prior to centrifugation. The RNA pellet was then washed with 75% ethanol (Sigma-Aldrich) and centrifuged before resuspension in nuclease-free water. Total RNA (10 µg) was DNase digested using the RNase-Free DNase kit (Qiagen). After the DNase digest RNA was cleaned-up using the RNeasy Mini Kit (Qiagen), as per the manufacturer’s instructions.

### RNA-seq analysis

RNA was extracted from tissues according to the protocol described above. Sample quality was analysed on an Agilent Tapestation (Agilent, Cheadle, Greater Manchester, UK). RNA libraries were then prepared using the SMARTer Stranded Total RNA-Seq Kit v2—Pico Input Mammalian (Takara, London, UK). The average library size (including adapters) was 360 bp. Samples were analysed for 50 bp paired end reads on a Novaseq 6000 sequencer (Illumina, Cambridge, Cambs, UK). Total RNA sequencing data was processed using nf-core/rnaseq v3.12.0 (10.5281/zenodo.1400710) of the nf-core collection of workflows [25]. GRCm38 was used as mouse reference genome. The differential expression analysis was performed using the nf-core/differentialabundance vx.x.x (10.5281/zenodo.7568000). The pipeline was configured with default parameters, unless otherwise specified, and executed with Nextflow v23.04.4 [26].

### Cell culture

Human pulmonary microvascular endothelial cells (PMVECs) were purchased from Promocell (Heidelberg, Germany) and maintained in EGM2-MV media (Promocell) with 2% FBS and antibiotic/antimycotic (A/A; Invitrogen) as per the supplier’s instructions. Human pulmonary artery smooth muscle cells (PASMCs) were isolated from vessel segments (5–8 mm diameter) that were cut to expose the luminal surface. The endothelium was removed by gentle scraping with a scalpel blade and the media then peeled away from the underlying adventitial layer. The medial explants were cut into 4- to 9-mm^2^ sections, plated into T25 flasks, and allowed to adhere for 2 h before addition of DMEM (Invitrogen) containing 20%(v/v) foetal bovine serum (FBS; Invitrogen) and A/A. The Royal Papworth Hospital ethical review committee approved the use of the human tissues (Ethics Ref: 08-H0304-56 + 5) and informed consent was obtained from all subjects. All cells were used between passages 4–8. Where indicated cells were treated with human recombinant BMP2, BMP9 or BMP10 (All R&D Systems). Treatment doses and times are indicated in the relevant figure legend.

### RNA extraction from cells

Total RNA was extracted from cells using RNeasy Mini Kit buffers (Qiagen) and EconoSpin® All-In-One Mini Spin Columns (Epoch Life Science, Sugarland, TX, USA) following the manufacturer’s instructions. On-column DNase (Qiagen) digest was performed as per the manufacturer’s instructions.

### Quantitative reverse transcription-PCR (QPCR)

Equal amounts of RNA (~ 1 μg) were reverse transcribed into cDNA using a High-Capacity Reverse Transcriptase kit (Thermo Fisher Scientific). Reactions for quantitative PCR were set up in MicroAmp® Optical 384-Well Reaction Plates (Thermo Fisher Scientific) using 2 μl/well cDNA, 5 μl/well PowerUp™ SYBR™ Green master mix, 1.8 µl/well premixed primer sets (final concentration in mix = 200 nM) and 1.2 μl/well DEPC-treated water. QPCR was run on a QuantStudio 6 Flex Real-Time PCR System (Thermo Fisher Scientific). Amplification reactions were activated with 2 min incubation at 50 °C followed by 2 min at 95 °C. This was followed by 50 amplification cycles of 15 s denaturation at 95 °C, 15 s annealing at 55 °C and 1 min extension at 72°C. Melt curve analysis was performed to rule out nonspecific amplification, and no-template controls were included. Primers were designed using Primer-BLAST (http://www.ncbi.nlm.nih.gov/tools/primer-blast/), and primer efficiency confirmed before use (Supplementary Table 1). Qiagen primers were used to detect human *EDN1*, *ITGA6*, *MYH11*, *SYT15* and *SMAD6* and mouse *Myh11*, *Rbp3* and *Syt15*. The relative expression levels of target genes were calculated using the 2^-(△△Ct) method by normalizing to the relevant housekeeping genes as detailed in the figure legends. Differences in gene expression are presented as the fold change relative to control. Relative expression of target genes in different cell lines or mouse tissues was determined as 2^-(CT^target^ -CT^housekeeping^).

### RNA-seq data processing and unsupervised machine learning

R/R-studio was used for statistical analysis. Transcript per million (TPM) data for RNA-seq identified genes in 356 PAH patients and 67 healthy controls was examined in pre-processed RNA-seq data [27, 28]. Survival analysis was performed using R package ‘Survival’ (v3.7) on the clustered patients. Kaplan–Meier survival curves from diagnosis were calculated using census date 01.07.2022 with right censoring, along with Cox proportional hazard models adjusting for age at diagnosis and sex. GitHub—https://github.com/RJD58/BMP9-KO-Dunmore.git

### Ethics

Samples data utilised from the UK National Cohort Study of Idiopathic and Heritable Pulmonary Arterial Hypertension were collected following informed consent (clinicaltrials.gov NCT01907295; UK REC Ref. 13/EE/0203) and/or the Sheffield Teaching Hospitals Observational Study of Pulmonary Hypertension, Cardiovascular and Other Respiratory Disease (UK REC Ref 18/YH/0441).

### Statistical analysis

Data are presented as mean ± S.E.M. and are analysed using GraphPad Prism 10 (GraphPad Software, Boston, MA, USA). Data were analysed by one-way analysis of variance (ANOVA) with post hoc Tukey’s honestly significant difference (HSD) analysis, two-way ANOVA followed by multiple comparisons using Tukey’s post hoc tests, or an unpaired two-tailed Student’s *t*-test as indicated. A value of *P* ≤ 0.05 was considered statistically significant.

## Results

### BMP9 knockout mice exhibit reduced pulmonary vessel muscularisation at baseline.

**Fig. 1:**
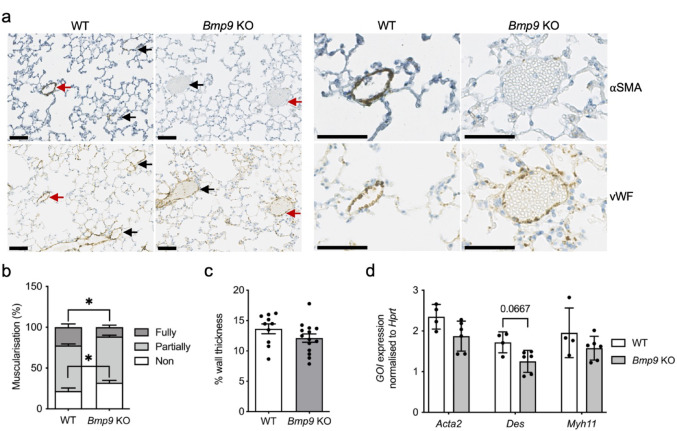
BMP9 deficiency reduces smooth muscle coverage. **a** *Left panel:* Serial lung sections immunostained with α-smooth muscle actin (αSMA) or von Willebrand factor (vWF) shown in lower magnification with serial section vessels labelled with arrows and, *Right panel:* Higher magnification of vessels (labelled with red arrows in the left panel) indicating αSMA and vWF staining. Scale bar = 50 μm. **b** Quantification of non, partially, or fully-muscularised vessels as a percentage of arteries associated with alveolar ducts in wild type (WT; n = 11) and *Bmp9* KO (n = 17) lungs. 20 arteries were counted per animal. **c** Wall thickness was evaluated by identifying small arteries (< 100 μm) proximal to the terminal epithelial bronchioles. Diameter was measured and then wall thickness measurements were assessed at four different positions of the artery, with a minimum of 10 arteries assessed in each lung section. **d** RNA was isolated from WT (n = 4) and *Bmp9* KO (n = 6) mice lungs. *Acta2*, *Des* and *Myh11* gene expression was normalised against *Hprt*. **b** Two-way ANOVA. (**c** and **d**) Unpaired t-test. *P ≤ 0.05. Error bars represent mean ± S.E.M

A previous study showed that *Gdf2*^*−/−*^ (*Bmp9* KO) mice exposed to chronic hypoxia have reduced right ventricular systolic pressures (RVSP) and reduced vessel remodelling in lungs [19]. We sought to determine whether *Bmp9* KO mice had any pulmonary vessel abnormalities at baseline. Reduced *Gdf2* expression was confirmed in the livers from the *Bmp9* KO mice compared to the WT mice (Supplementary Fig. 1a). RVSP, heart weight and spleen weight were similar in *Bmp9* KO mice and WT littermates (Supplementary Fig. 1b, c and d). Histological phenotyping of muscularisation in *Bmp9* KO and WT mice lungs was conducted using α-smooth muscle actin (αSMA) staining. In *Bmp9* KO mice, increased non-muscularised and decreased fully-muscularised vessels were observed compared to WT mice (Fig. 1a and b). However, we observed that pulmonary artery wall thickness was unaffected in the *Bmp9* KO mice (Fig. 1c). We examined the expression of genes associated with smooth muscle cells in the *Bmp9* KO mouse lungs. The genes encoding αSMA (*Acta2*), desmin (*Des*) and smooth muscle myosin heavy chain 11 (*Myh11*) were partially decreased but this was not statistically significant (Fig. 1d). There was also no difference in αSMA or desmin lung protein expression in WT or *Bmp9* KO mice (Supplementary Fig. 1e and f).

### RNA-seq identified genes associated with BMP9 knockout are partially reversed by BMP9 treatment.

**Fig. 2:**
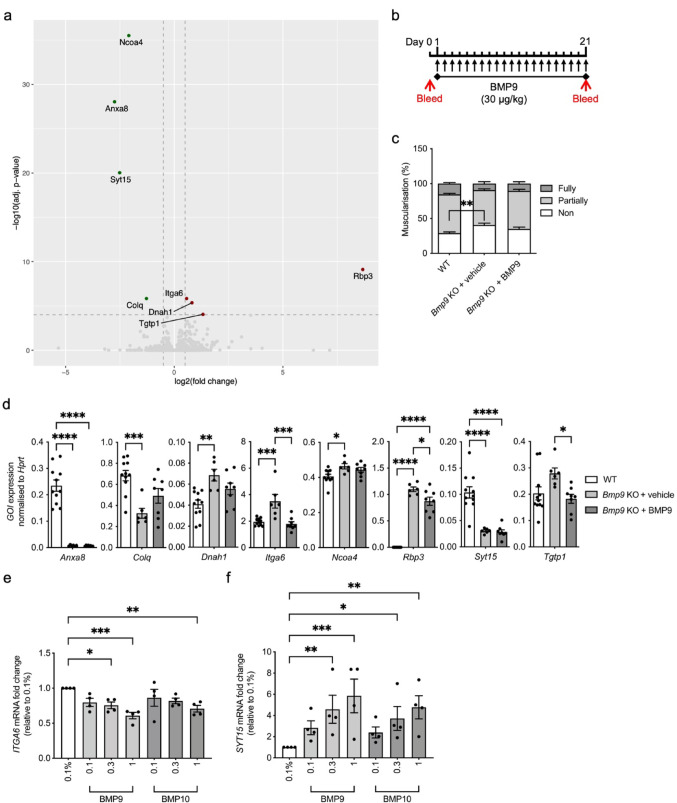
RNA sequencing of *Gdf2*^*−/−*^ lungs identifies genes associated with BMP9 loss. **a** RNA was isolated from wild type (WT; n = 4) and *Bmp9* KO (n = 6) mice lungs. Following RNA libraries preparation, samples were analysed for 50 bp paired end reads on a Novaseq 6000 sequencer (Illumina). Volcano plot of differentially expressed genes in *Bmp9* KO versus WT after fitting linear models and adjusting P values for multiple testing. **b** Schematic of treatment regime. WT and *Bmp9* KO mice were administered daily for 3-weeks with 0.03 mg/kg recombinant human BMP9 or vehicle control. Mice were bled at the beginning and end of treatment regime to check BMP9 levels. **c** Lung sections were immunostained with α-smooth muscle actin (αSMA). Quantification of non-, partially, or fully-muscularised vessels as a percentage of arteries associated with alveolar ducts in WT (n = 11), *Bmp9* KO plus vehicle (n = 7) and *Bmp9* KO plus BMP9 (n = 8) mice. 20 arteries were counted per animal. **d** RNA was isolated from WT (n = 11), *Bmp9* KO plus vehicle (n = 6/7) and *Bmp9* KO plus BMP9 (n = 6/8) mice lungs. Gene expression *Anxa8*, *Colq*, *Dnah1*, *Itga6*, *Rbp3*, *Syt15* and *Tgtp1* was normalised against the housekeeping gene, *Hprt*. **e** and **f** Human pulmonary microvascular cells (PMVECs; n = 4 biological replicates) were serum-starved (0.1%) overnight prior to treatment with BMP9 or BMP10 (0.1, 0.3, 1 ng/ml) for 8 h. Gene expression of *ITGA6* (**e**) and *SYT15* (**f**) was measured using qPCR, normalised to 2 housekeeping genes (*B2M* and *HPRT*). (**c**) Two-way ANOVA. (**d**, **e,** and **f**) One-way ANOVA. *P ≤ 0.05, **P ≤ 0.01, ***P ≤ 0.001, ****P ≤ 0.0001. Error bars represent mean ± S.E.M

Next, we applied RNA sequencing to interrogate the differentially expressed genes in the lungs of *Bmp9* KO mice compared to WT mice littermates (Fig. 2a and Supplementary Table 2). Surprisingly, only a small number of differentially expressed genes (DEGs) significantly changed (Fig. 2a). *Anxa8*, *Colq*, *Ncoa4* and *Syt15* were downregulated in *Bmp9* KO mice, whereas *Dnah1*, *Itga6*, *Rbp3* and *Tgtp1* were upregulated (Fig. 2a).

Using a separate animal cohort, we validated the phenotype and transcriptional changes observed in the *Bmp9* KO mice and investigated whether treatment with BMP9 was sufficient to reverse them. *Bmp9* KO mice were dosed daily with recombinant human BMP9 at 0.03 mg/kg for 3 weeks (Fig. 2b). Reduced circulating BMP9 in the serum of *Bmp9* KO mice was confirmed by ELISA, but increased BMP9 levels were not detectable in most mice following recombinant BMP9 treatment (Supplementary Fig. 2a). Liver *Gdf2* mRNA expression was significantly reduced in *Bmp9* KO mice, and unaffected by BMP9 treatment (Supplementary Fig. 2b). As previously demonstrated, *Bmp9* KO mice had more non-muscularised vessels compared to WT controls, whereas *Bmp9* KO mice treated with BMP9 were unaffected (Fig. 2c and Supplementary Fig. 2c). As shown in the RNA-seq, *Anxa8*, *Colq* and *Syt15* were downregulated, and *Dnah1*, *Itga6*, *Rbp3* and *Tgtp1* were upregulated in *Bmp9* KO mice (Fig. 2d). Only *Ncoa4*, was not validated compared to the lung RNA-seq analysis (Fig. 2d). Both *Itga6* and *Tgtp1* upregulation were reversed by recombinant BMP9 treatment, and the *Colq*, *Dnah1* and *Rbp3* changes partially reversed (Fig. 2d). As previously observed, the smooth muscle associated genes, *Acta2*, *Des* and *Myh11*, were unchanged (Supplementary Fig. 2d). Interestingly, *Smad6* expression was significantly elevated by BMP9 treatment but not reduced by BMP9 knockout (Supplementary Fig. 2e). The potent vasodilator adrenomedullin (*Adm*) is reported to be elevated, whereas the vasoconstrictor peptide endothelin-1 (*Edn1*) is substantially reduced in *Bmp9* KO mice in chronic hypoxia [19]. In our cohort of *Bmp9* KO mice under normoxic conditions neither *Adm* nor *Edn1* were changed, although BMP9 treatment did reduce *Adm* levels in agreement with the findings in BMP9 treated pulmonary artery endothelial cells (Supplementary Fig. 2f) [17].

We also examined the expression of the human homologues of the DEGs identified by RNA-seq in PASMCs and PMVECs in response to BMP9 stimulation, except *Tgtp1*, which is a mouse only gene. *ANXA8*, *COLQ*, *DNAH1* and *RBP3* were not expressed by PASMCs or PMVECs (data not shown). In PASMCs, BMP target genes, *ID1* and *ID2* were induced as expected by 10 ng/ml BMP2 and consistently activated by 3 and 30 ng/ml of BMP9 and BMP10 treatment for 8 h (Supplementary Fig. 3a and b). *ITGA6* and *SYT15* were not regulated by BMPs in PASMCs under these conditions (Supplementary Fig. 3c and d). As expected, in pulmonary microvascular endothelial cells (PMVECs), *ID1* and *ID2* were upregulated at much lower concentrations of BMP9 and BMP10 (Supplementary Fig. 3e and f) [29]. In PMVECs *ITGA6* was downregulated whereas *SYT15* was significantly elevated by BMP9 and BMP10 treatments at 0.3 and 1 ng/ml, opposing the effects seen in the *Bmp9* KO mice (Fig. 2e, f).

### Low ITGA6 expression associates with survival in PAH.

**Fig. 3:**
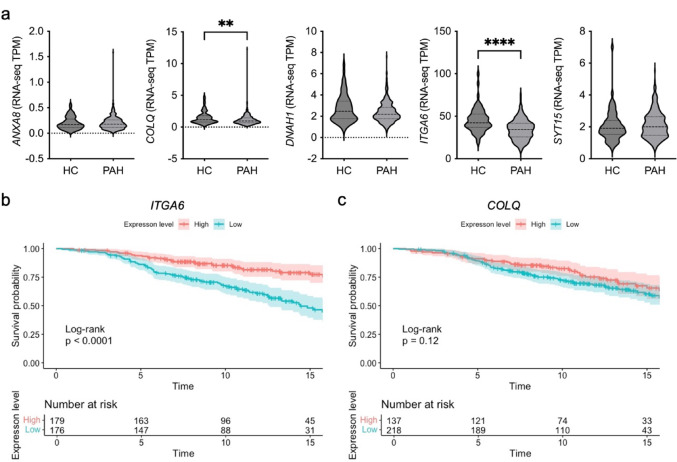
Differential expression of genes identified in the *Bmp9* KO RNA-seq analysis in PAH patients vs healthy controls. **a** Levels of *ANXA8*, *COLQ*, *DNAH1*, *ITGA6* and *SYT15* measured in the UK PAH cohort study using RNA-seq divided into healthy controls (HC; n = 67), and PAH (n = 356; IPAH = 285; *BMPR2* mutations-PAH = 71). TPM = transcript per million). Median and standard deviation values are detailed in Supplementary Table 3. **b** and **c** ﻿Kaplan–Meier survival analysis for patients stratified by mean gene expression. High and low expression of *COLQ* and *ITGA6* was divided above and below the mean. Statistical analysis was performed using pairwise log-rank test. Time = years. **a** Unpaired t-test. **P ≤ 0.01, ****P ≤ 0.0001

We examined the expression of these putative biomarkers in an RNA-seq dataset of peripheral blood from 356 idiopathic, heritable, and drug-induced PAH patients and 67 healthy controls [27, 28]. The relative transcripts per million (TPM) for *RBP3* was below 0.005, so this was excluded from analysis. Both *COLQ* and *ITGA6* were downregulated in PAH patients (Fig. 3a and Supplementary Table 3). No changes were observed for *ANXA8*, *DNAH1* or *SYT15* (Fig. 3a and Supplementary Table 3). Kaplan Meier survival curves demonstrated significantly reduced transplant free survival in patients with reduced *ITGA6* (*ITGA6* split at mean into high > 34.2TPM > low; *p* < 0.0001), which was confirmed with Cox proportional hazard modelling after correction for sex and age at diagnosis (hazard ratio 0.73, *p* = 0.003) (Fig. 3b and Supplementary Fig. 4a). No mortality association was observed for *COLQ* (Fig. 3c and Supplementary Fig. 4b).

### Chronic administration of a BMP9 neutralising antibody does not impact on vessel muscularisation or gene expression in wild type mice.

**Fig. 4:**
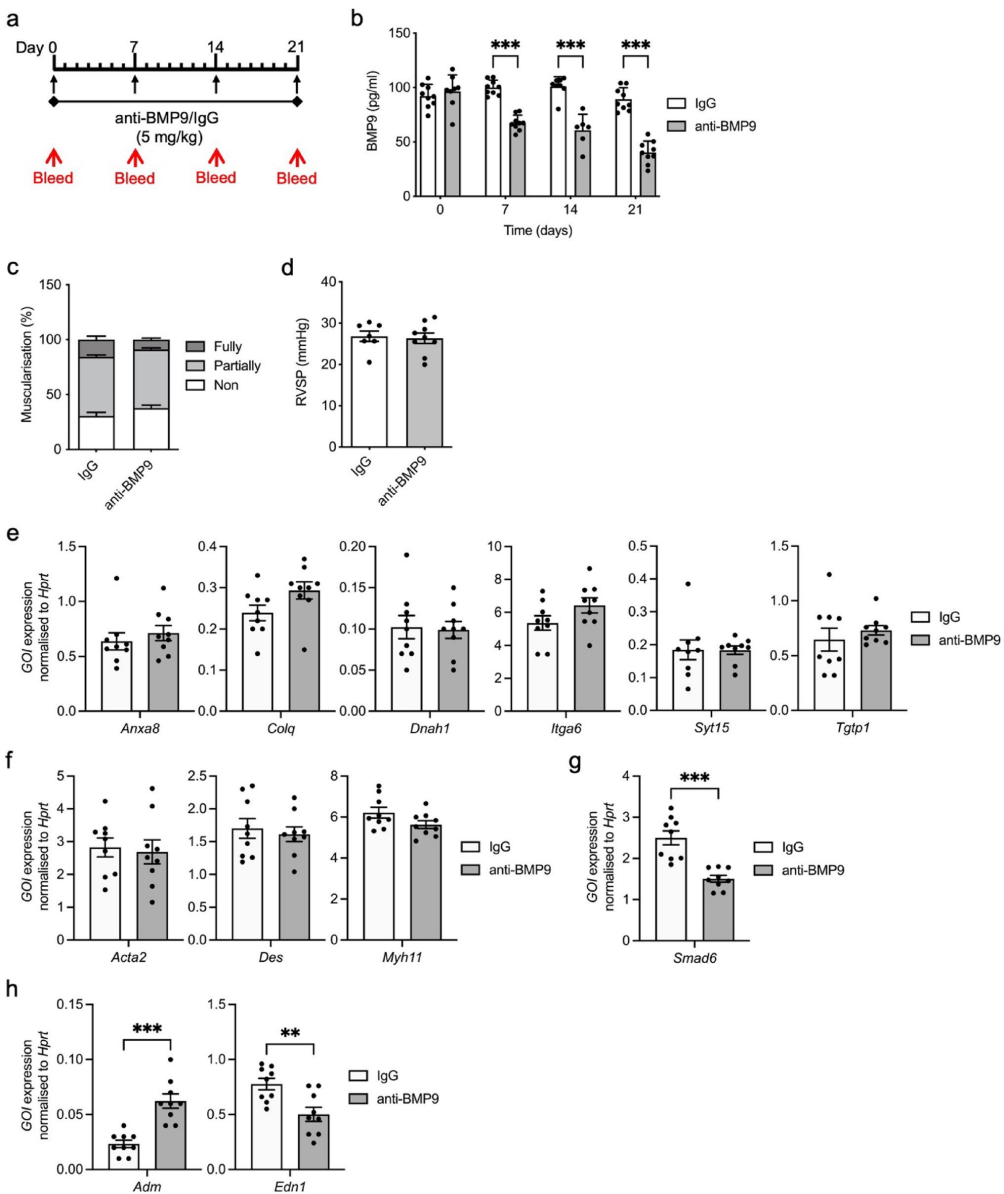
Anti-BMP9 treatment causes distinct transcriptional changes. **a** Schematic of treatment regime. Wild type (WT) mice were administered weekly for 3-weeks with 5 mg/kg BMP9 antibody (anti-BMP9) or equivalent volume of mouse IgG2B (IgG) isotype as a control group. Mice were bled every week to check BMP9 levels. Relevant tissue was collected after 3-weeks. **b** WT mice were administered weekly for 3-weeks with 5 mg/kg BMP9 antibody (anti-BMP9) or equivalent volume of mouse IgG2B (IgG) isotype as a control group. Mice were bled every week to check BMP9 levels. Mice treated with IgG (n = 8/9) or anti-BMP9 (n = 6–9) were bled every week to check BMP9 levels in serum using a BMP9 specific ELISA. **c** Quantification of non-, partially, or fully-muscularised vessels as a percentage of arteries associated with alveolar ducts in IgG (n = 9) and anti-BMP9 (n = 9) treated mice. 20 arteries were counted per animal. **d** RVSP was measured in IgG (n = 7) and anti-BMP9 (n = 9) mice. (**e**—**h**) RNA was isolated from lungs from WT mice treated with IgG (n = 9) or anti-BMP9 (n = 9) mice. Gene expression was normalised against the housekeeping gene, *Hprt*. **e** *Anxa8*, *Colq*, *Dnah1*, *Itga6*, *Syt15* and *Tgtp1* expression. **f** *Acta2*, *Des* and *Myh11* expression. **g** *Smad6* expression. **h** *Adm* and *Edn1* expression. **b** One-way ANOVA. **g** and **h** Unpaired t-test. **P ≤ 0.01, ***P ≤ 0.001. Error bars represent mean ± S.E.M

Chronic administration of a neutralising monoclonal BMP9 antibody reduces RVSP and vessel remodelling in chronic hypoxia [19]. We wondered whether a BMP9 neutralising antibody or IgG control administered weekly to wild type mice for 21 days would initiate reduced muscularisation (Fig. 4a). Treatment with anti-BMP9 at 5 mg/kg effectively reduced circulating BMP9 compared with the IgG control as measured by BMP9 specific ELISA (Fig. 4b). Vessel remodelling assessed by αSMA staining showed no statistical change in non-muscularised vessels (Fig. 4c and Supplementary Fig. 5). RVSP was unaffected by anti-BMP9 administration (Fig. 4d).

As before, we assessed the expression of genes associated with BMP9 knockout or treatment. There were no changes in the DEGs identified by the *Bmp9* KO mice RNA-seq (Fig. 4e). Smooth muscle markers *Acta2*, *Des* and *Myh11* were unaffected by the anti-BMP9 treatment (Fig. 4f). Expression of *Smad6* was reduced, although this did not match our observations in the *Bmp9* KO mice (Fig. 4g). Anti-BMP9 treatment elevated *Adm* and reduced *Edn1* expression (Fig. 4h), which correlates with the observations in *Bmp9* KO mice exposed to chronic hypoxia [19].

### Tamoxifen treatment of *Bmp9* KO and dKO mice causes smooth muscle associated transcriptional changes.

So far, we have shown that BMP9 knockout mice had reduced muscularisation associated with a select number of DEGs. Also, anti-BMP9 treatment had no effect on these. We therefore wondered whether this discrepancy between BMP9 knockout and anti-BMP9 treatment could be explained by BMP10 compensation.

**Fig. 5:**
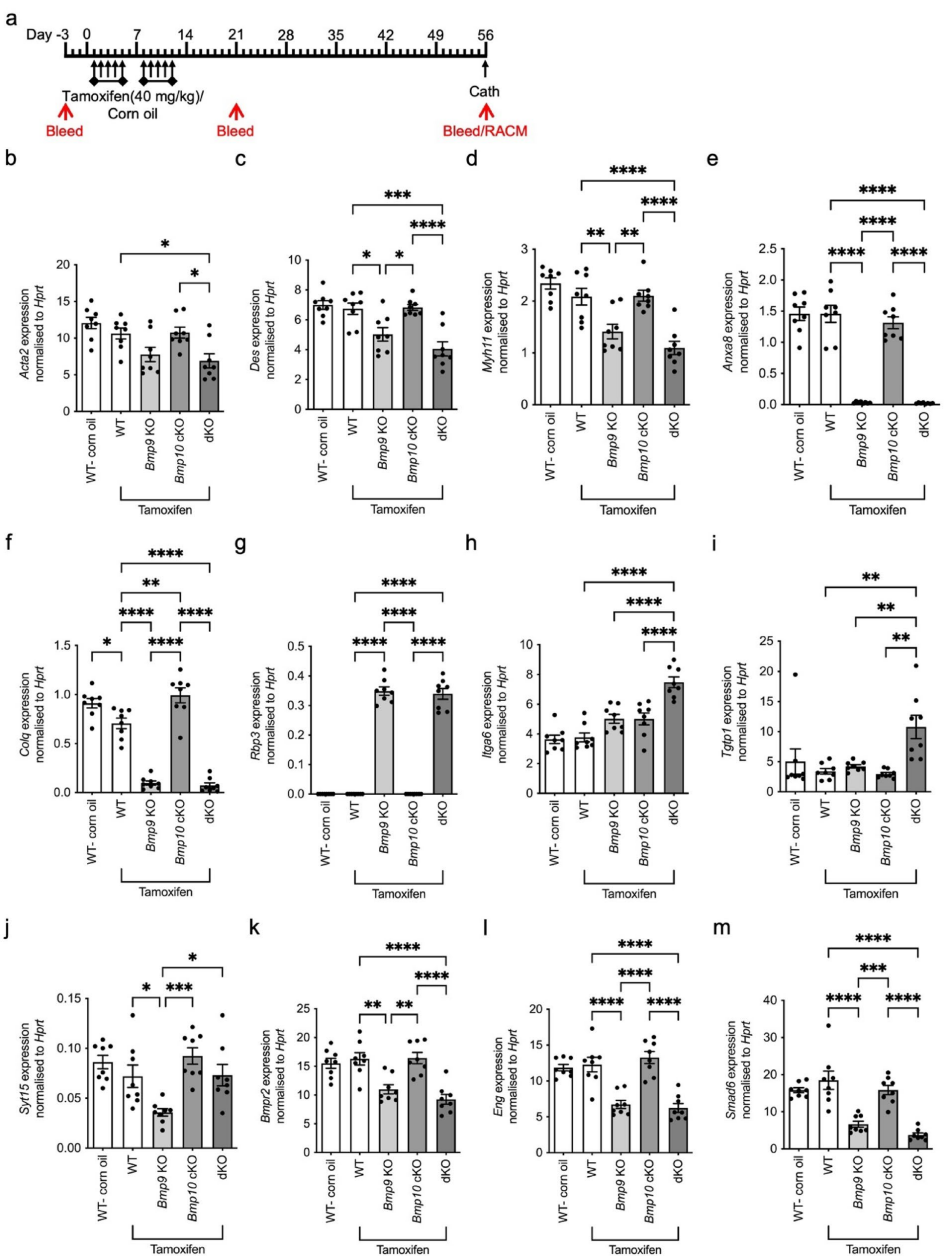
*Bmp9* KO and double knockout mice treated with tamoxifen exhibit reduced smooth muscle associated gene expression. **a** Schematic of treatment regime. *Bmp10*^*fl/fl*^ (WT), *Bmp10*^*fl/fl*^x*Gdf2*^*−/−*^ (*Bmp9* KO), *Bmp10*^fl/fl^xRosa26^Cre−ERT^ (*Bmp10* cKO) and *Bmp10*^fl/fl^xRosa26^Cre−ERT^x*Gdf2*^*−/−*^ (dKO) mice were treated with tamoxifen once a day for five days with a two-day recovery period followed by a further 5 days at a dose of 40 mg/kg. As a vehicle control, WT mice were administered corn oil for the same period. Mice then underwent right heart catheterisation on day 56. Mice were also bled at day -3, 21 and 56 to assess BMP9 levels. Right atrium was also taken at day 56 to generate BMP10 conditioned media (RACM). Relevant tissue was collected on day 56. **b–l** RNA was isolated on day 56 from lungs of WT (corn oil; n = 8), WT (tamoxifen; n = 8), *Bmp9* KO (tamoxifen; n = 8), *Bmp10* cKO (tamoxifen; n = 8) and dKO (tamoxifen; n = 8). Gene expression was normalised against the housekeeping gene, *Hprt*. *Acta2* (**b**), *Des* (**c**), *Myh11* (**d**), *Anxa8* (**e**), *Colq* (**f**), *Rbp3* (**g**), *Itga6* (**h**), *Tgtp1* (**i**), *Syt15* (**j**), *Bmpr2* (**k**), *Eng* (**l**) and *Smad6* (**m**) gene expression. **b**–**m** One-way ANOVA. *P ≤ 0.05, **P ≤ 0.01, ***P ≤ 0.001, ****P ≤ 0.0001. Error bars represent mean ± S.E.M

To examine the effect of BMP9 and BMP10 the following mice were generated: *Bmp10*^*fl/fl*^ (WT), *Bmp9* KO, *Bmp10*^fl/fl^xRosa26^Cre−ERT^ (*Bmp10* cKO) and *Bmp9* KOx*Bmp10*^fl/fl^xRosa26^Cre−ERT^ (dKO) (see relevant methods section). To induce Cre recombination and conditional knockout of BMP10 in mice harbouring the Cre-ERT allele, all cohorts were treated with tamoxifen by intraperitoneal injection, with their Cre-negative littermates included as controls (Fig. 5a). Control mice harbouring *Bmp10*^*fl/fl*^, but negative for Cre-ERT were dosed with an equivalent volume of corn oil (Fig. 5a). As previously shown, knockout of BMP9 was confirmed by ELISA of serum (Supplementary Fig. 6a). To confirm reduced BMP10, an ELISA measuring the growth factor domain (GFD) of BMP10 was used. Specificity was assessed by measuring recombinant BMP10 GFD, prodomain BMP10 (proBMP10) and a non-cleavable proBMP10 variant (R313A) that we previously manufactured [24]. The EC50 for GFD and proBMP10 was 714 and 475 pg/ml, respectively (Supplementary Fig. 6b). As expected, the non-processable proBMP10 (R131A) had an EC50 of 6538 pg/ml (Supplementary Fig. 6b). BMP10 knockout was confirmed in conditioned media from right atrial cultures (Supplementary Fig. 6c). As previously observed, serum BMP9 levels were reduced by BMP10 knockout (Supplementary Fig. 6a) [30].

It was previously reported that smooth muscle markers such as *Acta2* and *Myh11* were downregulated in dKO mice compared to BMP9 knockout mice [31]. In this cohort of mice treated with tamoxifen, we saw *Acta2*, *Des* and *Myh11* downregulated in the dKO mice, which agrees with the previous study. However, these genes were also reduced in the *Bmp*9 KO mice after tamoxifen treatment compared to wild type mice treated with tamoxifen (Fig. 5b–d).

Of the RNA-seq targets, mice lacking BMP9 and treated with tamoxifen had reduced *Anxa8*, *Colq* and *Rbp3* which weren’t further altered in the dKO mice, suggesting BMP9 loss was the driving factor (Fig. 5e–g). Double loss of BMP9 and 10 significantly enhanced *Itga6* and *Tgtp1* gene expression (Fig. 5h, i). As before, *Syt15* expression was significantly downregulated in the *Bmp9* KO mice but was restored in the dKO (Fig. 5j). Interestingly, in this cohort of mice, *Dnah1* was unaffected (Supplementary Fig. 6d). As previously reported, *Edn1* was reduced in *Bmp9* KO mice and dKO mice (Supplementary Fig. 6e), but *Adm* expression was unchanged (Supplementary Fig. 6f). Intriguingly, BMP target genes *Bmpr2*, *Eng* and *Smad6* were significantly downregulated in dKO mice and tamoxifen treated *Bmp9* KO mice (Fig. 5k–m). We confirmed that *Bmpr2*, *Eng* and *Smad6* expression was unaffected in *Bmp9* KO mice without tamoxifen (Supplementary Fig. 6g).

**Fig. 6:**
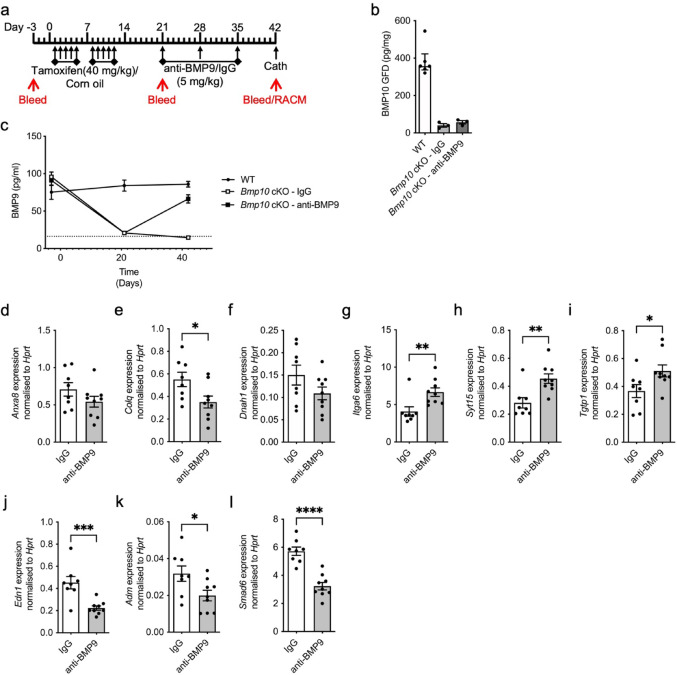
Transcriptional changes in conditional knockout mice treated with anti-BMP9. **a** Schematic of treatment regime. *Bmp10*^fl/fl^xRosa26^Cre−ERT^ (*Bmp10*-cKO) were treated with tamoxifen once a day for five days with a two-day recovery period followed by a further 5 days at a dose of 40 mg/kg. As a vehicle control *Bmp10*^*fl/fl*^ (WT) mice were administered corn oil for the same period. On day 21 mice were dosed weekly for 2-weeks with 5mg/kg BMP9 antibody (anti-BMP9) or equivalent volume of mouse IgG2B (IgG) isotype as a control. Mice then underwent right heart catheterisation on day 42. Mice were also bled at day -3, 21 and 42 to assess BMP9 levels. Right atrium was also taken at day 42 to generate BMP10 conditioned media (RACM). Relevant tissue was collected on day 42. **b** Conditioned media from right atria collected at day 42 from WT (corn oil; n = 6), *Bmp10* cKO – IgG (tamoxifen; n = 3) and *Bmp10* cKO—anti-BMP9 (tamoxifen; n = 3) mice was assayed for BMP10 levels using a BMP10 growth factor domain (GFD) specific ELISA. **c** Serum from WT (corn oil; n = 6), *Bmp10* cKO—IgG (n = 9) and *Bmp10* cKO—anti-BMP9 (*Bmp10* cKO—anti-BMP9; n = 9) mice bled at day -3, 21 and 42 were assayed for BMP9 levels using a BMP9 specific ELISA. **d**–**l** RNA was isolated on day 45 from lungs of *Bmp10* cKO (IgG; n = 8) and *Bmp10* cKO (anti-BMP9; n = 9). Gene expression was normalised against the housekeeping gene, *Hprt*. *Anxa8* (**d**), *Colq* (**e**), *Dnah1* (**f**), *Itga6* (**g**) *Syt15* (**h**), *Tgtp1* (**i**), *Edn1* (**j**), *Adm* (**k**) and *Smad6* (**l**) expression. (**e**, **g**, **h**, **i**, **j**, **k,** and **l**) Unpaired t-test. *P ≤ 0.05, **P ≤ 0.01, ***P ≤ 0.001, ****P ≤ 0.0001. Error bars represent mean ± S.E.M

We also examined *Bmp10* cKO mice treated with anti-BMP9 to determine whether post-natal loss of BMP9 accounted for the substantial transcriptional changes in the presence of tamoxifen (Fig. 6a). ELISA confirmed loss of right atrial BMP10 in the *Bmp10* cKO mice (Fig. 6b). As previously observed, loss of BMP10 caused reduced serum BMP9 levels, although anti-BMP9 treatment increased BMP9 (Fig. 6c). Of the genes identified by RNA-seq—*Anxa8*, *Colq*, *Dnah1*, *Itga6* and *Tgtp1* were similarly regulated in the *Bmp10* cKO treated with anti-BMP9 compared to IgG-dosed mice (Fig. 6d–g, i). However, *Syt15* expression was reversed by anti-BMP9 treatment and BMP10 loss (Fig. 6h). Like dKO mice, *Edn1* was reduced by anti-BMP9 in the *Bmp10* cKO mice (Fig. 6j). Unlike the dKO study, *Adm* expression was reduced (Fig. 6k). Once again *Smad6* was downregulated by anti-BMP9 treatment (Fig. 6l). Supplementary Table 4 summarises selected relative gene expression changes across all animal models.

### Tamoxifen treatment of *Bmp9* KO and dKO mice causes tissue remodelling.

**Fig. 7:**
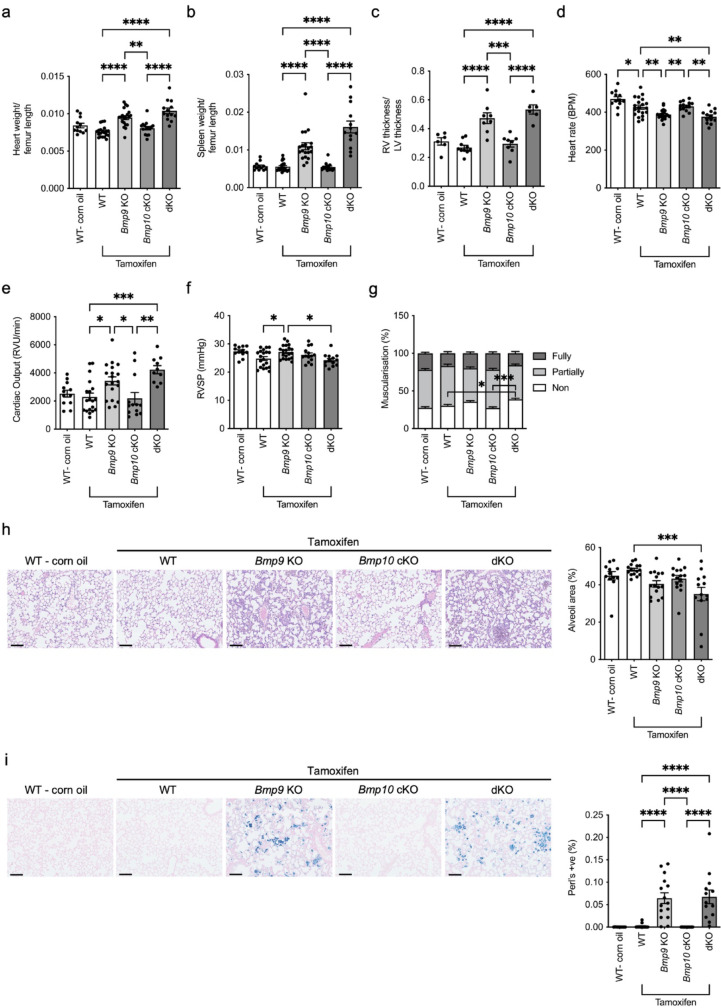
*Bmp9* KO and double knockout mice treated with tamoxifen exhibit extensive tissue remodelling. (**a**–**g**) *Bmp10*^*fl/fl*^ (WT), *Bmp10*^*fl/fl*^x*Gdf2*^*−/−*^ (*Bmp9* KO), *Bmp10*^fl/fl^xRosa26^Cre−ERT^ (*Bmp10* cKO) and *Bmp10*^fl/fl^xRosa26^Cre−ERT^x*Gdf2*^*−/−*^ (dKO) mice were treated with tamoxifen once a day for five days with a two-day recovery period followed by a further 5 days at a dose of 40 mg/kg. As a vehicle control, WT mice were administered corn oil for the same period. Mice then underwent right heart catheterisation on day 56. Relevant tissue was collected on day 56. **a** Heart weight was assessed as a ratio of femur length in WT (corn oil; n = 12), WT (tamoxifen; n = 20), *Bmp9* KO (tamoxifen; n = 20), *Bmp10* cKO (tamoxifen; n = 15) and dKO (tamoxifen; n = 13). **b** Spleen weight was assessed as a ratio of femur length in WT (corn oil; n = 12), *Bmp10*^*fl/fl*^ (tamoxifen; n = 20), *Bmp9* KO (tamoxifen; n = 20), *Bmp10* cKO (tamoxifen; n = 15), and dKO (tamoxifen; n = 13). **c** Ratio of right ventricle (RV) thickness and left ventricle thickness (LV) in WT (corn oil; n = 6), WT (tamoxifen; n = 10), *Bmp9* KO (tamoxifen; n = 8), *Bmp10* cKO (tamoxifen; n = 8) and dKO (tamoxifen; n = 6). **d** Heart rate was measured in WT (corn oil; n = 12), WT (tamoxifen; n = 20), *Bmp9* KO (tamoxifen; n = 20), *Bmp10* cKO (tamoxifen; n = 14) and dKO (tamoxifen; n = 13). **e** Measurement of cardiac output in WT (corn oil; n = 12), WT (tamoxifen; n = 19), *Bmp9* KO (tamoxifen; n = 19), *Bmp10* cKO (tamoxifen; n = 13) and dKO (tamoxifen; n = 10). **f** Right ventricular systolic pressure (RVSP) was measured in WT (corn oil; n = 12), WT (tamoxifen; n = 20), *Bmp9* KO (tamoxifen; n = 20), *Bmp10* cKO (tamoxifen; n = 14) and dKO (tamoxifen; n = 13). **g** Lung sections were immunostained with α-smooth muscle actin (αSMA). Quantification of non, partially, or fully-muscularised vessels as a percentage of arteries associated with alveolar ducts in WT (corn oil; n = 12), WT (tamoxifen; n = 15), *Bmp9* KO (tamoxifen; n = 15), *Bmp10*-cKO (tamoxifen; n = 15) and dKO (tamoxifen; n = 13) mice. 20 arteries were counted per animal. **h** Alveoli area was assessed in haematoxylin and eosin-stained lung sections. Percentage of counterstained tissue versus no staining of the whole lung area in WT (corn oil; n = 12), WT (tamoxifen; n = 15), *Bmp9* KO (tamoxifen; n = 15), *Bmp10*-cKO (tamoxifen; n = 15) and dKO (tamoxifen; n = 13) mice. (**i**) Lung sections were stained with Perl’s iron stain. Percentage of Perl’s positive cells in whole lung area from WT (corn oil; n = 12), WT (tamoxifen; n = 15), *Bmp9* KO (tamoxifen; n = 15), *Bmp10*-cKO (tamoxifen; n = 15) and dKO (tamoxifen; n = 13) mice. Scale bar = 100 μm. (**a**, **b**, **c**, **d**, **e**, **f**, **h** and **i**) One-way ANOVA. **g** Two-way ANOVA. *P ≤ 0.05, **P ≤ 0.01, ***P ≤ 0.001, ****P ≤ 0.0001. Error bars represent mean ± S.E.M

We examined the physiological changes previously reported to be associated with BMP9 and BMP10 loss [20]. Our dKO mice replicated previous findings of cardiomegaly, splenomegaly, and a significantly increased ratio of right ventricle thickness to left ventricle thickness, importantly, we found that these changes were also evident in tamoxifen treated *Bmp9* KO mice (Fig. 7a–c). Additionally, heart rate and cardiac output was also significantly reduced in the tamoxifen treated *Bmp9* KO mice (Fig. 7d, e). Furthermore, tamoxifen treated *Bmp9* KO mice had marginally elevated RVSP compared with the tamoxifen treated wild type control and dKO mice (Fig. 7f). In normoxic conditions, non-muscularised pulmonary vessels were significantly increased in dKO mice, with a trend but non-significant increase in *Bmp9* KO mice (Fig. 7g and Supplementary Fig. 7). No changes in muscularisation in the *Bmp10* cKO and WT mice treated with tamoxifen were observed (Fig. 7g and Supplementary Fig. 7). We also observed reduced alveoli space in the dKO mice (Fig. 7h). Double loss of BMP9 and BMP10 increases the presence of iron-laden macrophages [20]. However, we observed a dramatic increase in hemosiderosis in both the dKO and tamoxifen treated BMP9 knockout mice (Fig. 7i).

We also examined *Bmp10* cKO mice treated with anti-BMP9 to determine whether post-natal loss of BMP9 accounted for the substantial physiological changes. The observed changes in cardiomegaly, heart rate, cardiac output and RVSP were not reciprocated in the *Bmp10* cKO (Supplementary Fig. 8a, c, d, and e). However, loss of BMP10 and treatment with anti-BMP9 did cause splenomegaly (Supplementary Fig. 8b). No vascular remodelling was observed (Supplementary Fig. 8f and g).

**Fig. 8:**
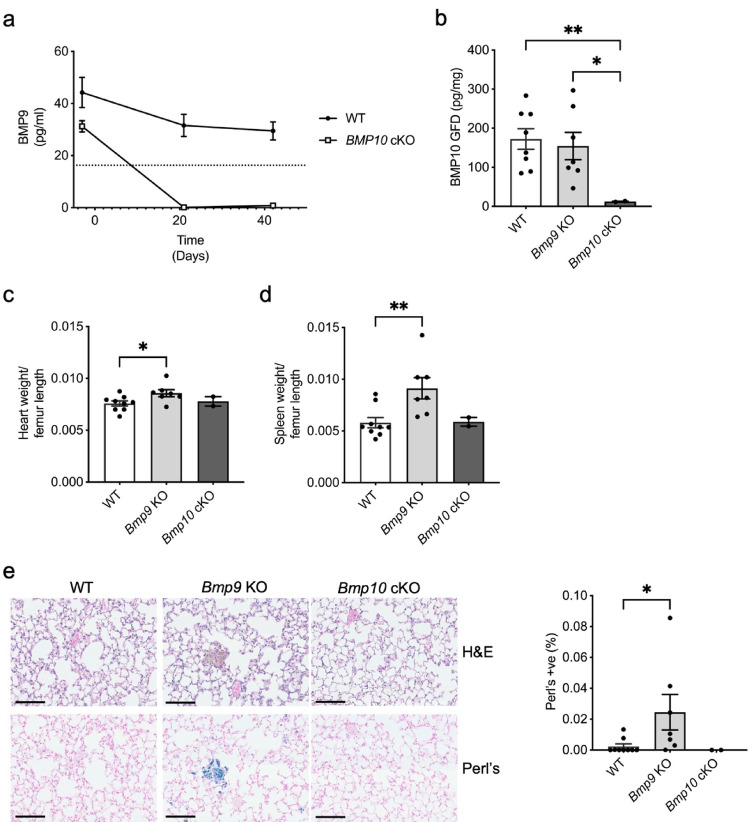
*Bmp9* KO mice treated with tamoxifen have cardiomegaly and splenomegaly. Wild type (WT; n = 9), *Bmp9* KO (n = 7) and *Bmp10*^fl/fl^xRosa26^Cre−ERT^ (*Bmp10* cKO; n = 2) mice were treated with tamoxifen once a day for five days with a two-day recovery period followed by a further 5 days at a dose of 40 mg/kg. Mice were also bled at day -3, 21 and 42 to assess BMP9 levels. Right atria were collected at day 42 for conditioned media culture. **a** Serum from WT (n = 8), *Bmp9* KO (n = 7) and *Bmp10* cKO (n = 2) mice bled at day -3, 21 and 42 were assayed for BMP9 levels using a BMP9 specific ELISA. BMP9 was undetectable in *Bmp9* KO mice. **b** Conditioned media from right atria collected at day 42 from WT (n = 8), *Bmp9* KO (n = 7) and *Bmp10* cKO (n = 2) mice were assayed for BMP10 levels using a BMP10 growth factor domain (GFD) specific ELISA. **c** Heart weight was assessed as a ratio of femur length in WT (n = 9), *Bmp9* KO (n = 7) and *Bmp10*-cKO (n = 2). **d** Spleen weight was assessed as a ratio of femur length in WT (n = 9), *Bmp9* KO (n = 7) and *Bmp10* cKO (n = 2). **e** Alveoli area was assessed using haematoxylin and eosin and lung sections were stained with Perl’s iron stain. Scale bar = 100 μm. **f** Percentage of Perl’s positive cells in whole lung area from WT (n = 9), *Bmp9* KO (n = 7) and *Bmp10*-cKO (n = 2) mice. **b**, **c,** and **d** One-way ANOVA. **f** Unpaired t-test. *P ≤ 0.05, **P ≤ 0.01. Error bars represent mean ± S.E.M

We could not rule out that the above findings were not due to Cre recombinase toxicity. We, therefore, treated wild type and *Bmp9* KO littermates with tamoxifen using the same dosing protocol (Figs. 5a, 6a) and included *Bmp10* cKO sentinels as controls. Serum BMP9 was undetectable in *Bmp9* KO mice and were reduced in the *Bmp10* cKO mice (Fig. 8a). Atrial BMP10 secretion was only reduced in the *Bmp10* cKO mice (Fig. 8b). Cardiomegaly and splenomegaly and hemosiderosis were only observed in BMP9 knockout mice, not in WT or Bmp*10* cKO mice (Fig. 8c–e), suggesting a gain of sensitivity to tamoxifen in the *Bmp9* KO mice.

## Discussion

Paradoxically, in humans, mutations leading to BMP9 deficiency cause PAH, whereas in mice, BMP9 loss protects against hypoxia induced PAH. Also, a significant decrease in smooth muscle coverage was reported in BMP9 knockout mice exposed to chronic hypoxia [19]. Of note in that study, under normoxic conditions distal vessel remodelling appeared to be reduced in *Bmp9* KO mice although it was not statistically significant and not highlighted [19]. Here we report the remodelling of the small pulmonary arteries with a loss of smooth muscle coverage in normoxic conditions. This is perhaps unsurprising given that *GDF2* mutations have been associated with pulmonary arterio-venous malformations [32].

We also replicated the findings that dKOs develop splenomegaly, hemosiderosis and severe cardiac changes with no differences observed in the *Bmp10* cKO mice. However, these changes were also observed in *Bmp9* KO mice treated with tamoxifen. Therefore, it is entirely possible that the phenotypes attributed to loss of both BMP9 and BMP10 in dKO mice is due to tamoxifen sensitivity on a background of BMP9 deficiency in *Bmp9* KO mice. Unsurprisingly, loss of BMP9 and BMP10 has been investigated in the context of HHT. Choi and colleagues examined the effects of *Bmp10* cKO and dKO, as well as *Bmp9* KO mice treated with tamoxifen on arteriovenous malformations, hypervascularisation and heart defects [33]. In this study, cardiomegaly was observed in both *Bmp10* cKO and dKO mice, but unlike our study not *Bmp9* KO mice. However, tamoxifen was administered postnatally on days 1–4, causing lethality by P7 and P10 for dKO and *Bmp10* cKO *mice*, respectively [33]. In our study, adult mice were dosed with tamoxifen once again highlighting the differing roles that BMP9 and BMP10 potentially play in vasculogenesis and angiogenesis.

The reduced muscularisation phenotype observed in *Bmp9* KO mice were not recapitulated in wild type mice treated with a BMP9 neutralising antibody. The lack of overlap between the two models is not unprecedented, as new-born mice treated with anti-BMP9 had significantly increased retinal vessel density, whereas BMP9 knockout mice had no impaired retinal vascularisation [34]. However, a limitation of our study is that anti-BMP9 appeared to only reduce BMP9 levels by approximately 50%, unlike the complete loss observed in the *Bmp9* KO mice. Additionally, we provide further evidence that the phenotypic changes observed in adult BMP9 and BMP10 knockout mice are not the same. Conditional adult BMP10 knockouts are induced by tamoxifen treatment to circumvent embryonic cardiac developmental defects caused by BMP10 loss [22]. Unlike BMP9 loss, mice deficient in BMP10 did not have reduced smooth muscle coverage. Again, similar differences were observed in a mammary cancer model where BMP9 deficiency resulted in increased tumour growth and lung metastases, with no effect observed in the inducible BMP10 knockout [35]. Also, a double knockout of BMP9 and BMP10 did not produce a stronger phenotype.

We reasoned that reduced mural cell association could be related to down regulation of smooth muscle marker transcription. However, RNA-seq analysis of lungs from wild type and *Bmp9* KO mice revealed only a select number of differentially expressed genes, none of which were associated with smooth muscle cell biology. Independent validation of the RNA-seq data revealed a select number of differentially expressed genes that matched the original RNA-seq dataset. All appear to be novel downstream targets of BMP9 in the mouse lung. The following genes were significantly downregulated by BMP9 loss. *ANXA8* encodes a protein that is part of the annexin family involved in Ca^2+^ and phospholipid binding, and HUVECs deficient in *ANXA8* exhibit impaired VEGF-A-dependent angiogenesis [36]. Surprisingly, in multiple biological replicates of pulmonary endothelial cells and smooth muscle cells in this study, *ANXA8* wasn’t highly expressed. *COLQ* encodes a collagen-like molecule that binds acetylcholinesterase in the skeletal muscle. COLQ plays an important functional role in the neuromuscular junction by binding the low-density lipoprotein receptor-related protein 4 (LRP4), which regulates the muscle-specific kinase (MuSK) [37]. *COLQ* mutations are associated with congenital myasthenic syndromes (CMS) with 2 case reports of associated PH [38, 39]. *SYT15* encodes a type 1 membrane protein of the synaptotagmin family of membrane trafficking proteins, members of which are reported to be involved in synaptic vesicle exocytosis and Ca^2+^-dependent trafficking [40]. Many synaptotagmins are well characterised for their role in neurotransmitter release, but the function of SYT15 is poorly understood [41]. Based upon tissue distribution and the lack of phospho-regions in the C2 domain it is thought that SYT15 is expressed non-neuronally and Ca^2+^-independent [40]. For the first time we show that *SYT15* is expressed by pulmonary endothelial cells and is upregulated by BMP9 and BMP10 treatment.

Of the genes upregulated in BMP9 knockout, we identified dynein axonemal heavy chain 1 (*DNAH1*) that encodes a subunit of the cytoskeletal motor protein, dynein. DNAH1 regulates sperm cilia and flagella function, and mutations cause multiple morphologic abnormalities of the flagella and male infertility due to impaired sperm motility (asthenozoospermia) [42]. Again, *DNAH1* expression was low in pulmonary endothelial and smooth muscle cells. Retinol binding protein 3 (*RBP3*) gene encodes a photoreceptor predominantly expressed by photosensitive tissue [43]. Recently the levels of RBP3 have been linked to the development of diabetic retinopathy [44]. Loss of BMP9 correlated with a dramatic increase in *Rbp3*. The T-cell specific mouse GTPase (*Tgtp1*) was significantly upregulated in BMP9 knockout mice. *Tgtp1* is reportedly a viral infection and interferon regulated gene in mice [45, 46]. *ITGA6* encodes the α6-integrin associated with cell–cell contacts, which binds either of the selective laminin receptors, ITGB1 or ITGB4 [47]. Cell and context specific changes in ITGA6 expression dictate its role in tumour biology with elevated levels associated with breast cancer, colorectal cancer and leukaemia [47]. BMP9 or BMP10 treatment down regulated *ITGA6* expression in pulmonary endothelial cells, with no effect on smooth muscle cells.

Another study using the BMP9 and BMP10 knockout reported decreased gene expression of *Acta2*, *Myh11* as well as transgelin (*Tagln*) and smoothelin (*Smtn*) in the aortas of double knockouts compared to *Bmp9* KO mice, although the expression in wild type mice was not compared [31]. As already discussed, we assessed the expression of smooth muscle markers *Acta2*, *Des* and *Myh11* in *Bmp9* KO mice with no changes observed. However, all these markers were significantly downregulated in both the dKO, and the *Bmp9* KO mice treated with tamoxifen. Again, this suggests that BMP9 loss is driving a sensitivity to tamoxifen treatment. It is worth noting that *Anxa8, Itga6* and *Smad6* have previously been reported as DEGs in RNA-seq analysis of lungs from double knockouts [20]. It is important to acknowledge that a limitation of our study and others is the reliance on RNA-seq datasets from whole lung homogenates. To mitigate this, single cell RNA-seq could help delineate the genes involved in the vascular remodelling observed in *Bmp9* KO mice, given the cellular heterogeneity in this process.

We investigated the expression of the above genes in PAH patient blood, showing that both *COLQ* and *ITGA6* were reduced, though only lower expression of *ITGA6* associated with poor survival. *Colq* expression was reduced by BMP9 loss in mice, although there was no further reduction by BMP10 loss. Although the patient data are counterintuitive as BMP9 and BMP10 loss increased *Itga6* expression, it could still provide insights into the role of integrins in the development and treatment of PAH. It is worth noting that the patient cohort we studied may not have reduced BMP9 and or BMP10 due to mutations, as only a small percentage of the PAH population show this phenotype [24]. Also, loss of BMP9 and BMP10 are not causal of PAH in experimental rodent models. Of note, integrin α5β1 is reported to be elevated in the remodelled pulmonary arteries of PAH patients, and pulmonary artery endothelial and smooth muscle cells [48]. Small molecule and antibody inhibition of α5β1 ameliorated established PAH in pre-clinical models [48].

We sought to determine the pathological, physiological, and transcriptional relevance of BMP9 knockout in the pulmonary vasculature. This study not only highlights the important role BMP9 plays in smooth muscle association under normal circumstances, but it also highlights several novel genes regulated by BMP9 treatment and loss. Genes such as *ANXA8*, *COLQ*, *ITGA6*, and *SYT15* could be potential biomarkers in diseases where BMP9 deficiency or treatment is evident. Our study also urges caution when interpreting cardiovascular and respiratory disease phenotypes when tamoxifen treatment is required to conditionally modify members of the BMP pathway. The use of the correct controls is therefore imperative in the understanding of this complex pathway.

## Supplementary Information

Below is the link to the electronic supplementary material.

Supplementary Material 1

## Data Availability

No datasets were generated or analysed during the current study.
