# Supplementary material for: BMP9 knockout impairs pulmonary vessel muscularisation and confers aberrant tamoxifen sensitivity
Source: Angiogenesis. 2025 Nov 12;29(1):5. doi: 10.1007/s10456-025-10017-5 (PMC12611992; doi:10.1007/s10456-025-10017-5)

**BMP9 knockout impairs pulmonary vessel muscularisation and confers aberrant tamoxifen sensitivity.**

Benjamin J. Dunmore^1^*, Stephen Moore^1^*, Rowena J. Jones^1^, Joshua Hodgson^1^, Kathryn Auckland^1^, Mark Southwood^2^, Nichola Figg^1^, Nobuhiro Kikuchi^1^, UK National Cohort Study of Idiopathic and Heritable PAH Consortium, ﻿the Uniphy Clinical Trials Network, Martin Bennett^1^, Allan Lawrie^3^, Christopher J. Rhodes^3^, Mark R. Toshner^1^, Stefan Gräf^1^**, Wei Li^1^**, Nicholas W. Morrell^1^**, Paul D. Upton^1^**.

**Supplementary Material**

**Supplementary Table 1. qPCR primer sequences.**

| Gene | Forward (5’) primer | Reverse (3’) primer |
| --- | --- | --- |
| Human *ID1* | GACGGCCGAGGCGGCATG | GGGGAGACCCACAGAGCACG |
| Human *ID2* | GACCCGATGAGCCTGCTATAC | GGTGCTGCAGGATTTCCATCT |
| Human *BACT* | GCACCACACCTTCTACAATGA | GTCATCTTCTCGCGGTTGGC |
| Human *B2M* | CTCGCGCTACTCTCTCTTTCT | CATTCTCTGCTGGATGACGTG |
| Human *HPRT* | GCTATAAATTCTTTGCTGACCTGCTG | AATTACTTTTATGTCCCCTGTTGACTGG |
| Mouse *Anxa8* | ACTCACCTGATGAGAGTGTTC | GCAAAGTAGCTGTGGACGTT |
| Mouse *Colq* | GCCCAGGAAGGAAGGGTAGA | AACCCTTAGAGCCCATCGGT |
| Mouse *Dnah1* | AAGCTGGACAAGAGGCGAAA | GGCACCATATGTCTCGAGGG |
| Mouse *Itga6* | CCTAACAGAATTGACCTCCGCCAGAAG | ACTGAACTCTCGATGACAACCCTGA |
| Mouse *Ncoa4* | GCTAAGTGTCTGGGTCGGTC | ATTCTGCTCACTGTTCCGGC |
| Mouse *Tgtp1* | ACTCCACACCTCATGTCCTCT | TCTGTATGGTAGAAGCTCAGCAG |
| Mouse *Acta2* | AGCCATCTTTCATTGGGATGGAG | CATGGTGGTACCCCCTGACA |
| Mouse *Des* | CGAGCAAAGGGGTTCTGAAGT | TAGCCTCGCTGACAACCTCT |
| Mouse *Bmpr2* | GGGTAAGCTCTTGCCGTCT | GTGGGAGATTGCGGGTTTAT |
| Mouse *Eng* | CACAACAGGTCTCGCAGAAA | GCTTGGATGCCTGAAGAGTC |
| Mouse *Smad6* | GGGTGAATTCTCAGATGCCAG | TGGTCGTACACCGCATAGAG |
| Mouse *Adm* | CCTGGACGAGCAGAACACAA | CTGAAATGTGCAGGTCCCGA |
| Mouse *Edn1* | TGCTATTGCTGATGGCCTCC | GGCCCAAAGTACCATGCAGA |
| Mouse *B2m* | GTATACTCACGCCACCCACC | TGGGGGTGAATTCAGTGTGAG |
| Mouse *Hprt* | CTTCCTCCTCAGACCGCTTT | ATCGCTAATCACGACGCTGG |

**Supplementary Table 2. Top 50 differentially expressed genes in Bmp9 KO mouse lungs compared to wild-type littermates.**

| **ensembl_gene_id** | **mgi_symbol** | **log2FoldChange** | **padj** |
| --- | --- | --- | --- |
| ENSMUSG00000056234 | Ncoa4 | -2.0913247 | 3.0455661539712e-36 |
| ENSMUSG00000021950 | Anxa8 | -2.7467436 | 9.03975229814665e-29 |
| ENSMUSG00000041479 | Syt15 | -2.5123398 | 9.24585361130352e-21 |
| ENSMUSG00000041534 | Rbp3 | 8.67601049 | 7.78669201052729e-10 |
| ENSMUSG00000027111 | Itga6 | 0.5695267 | 1.46862083633312e-6 |
| ENSMUSG00000057606 | Colq | -1.2799048 | 1.46862083633312e-6 |
| ENSMUSG00000019027 | Dnah1 | 0.8151387 | 4.44152073620907e-6 |
| ENSMUSG00000060586 | H2-Eb1 | 0.36767484 | 1.30424664895744e-5 |
| ENSMUSG00000078922 | Tgtp1 | 1.32317207 | 9.0880203106571e-5 |
| ENSMUSG00000021908 | Ncoa4-ps | 3.52256387 | 1.13224899171692e-4 |
| ENSMUSG00000021796 | Bmpr1a | -0.3268899 | 2.01654537287663e-4 |
| ENSMUSG00000060550 | H2-Q7 | 0.60194886 | 5.54533806091625e-4 |
| ENSMUSG00000087028 | Gm13387 | 0.94691242 | 6.3305402039508e-4 |
| ENSMUSG00000028268 | Gbp3 | 0.68238938 | 0.00119473 |
| ENSMUSG00000040253 | Gbp7 | 0.53373503 | 0.00119473 |
| ENSMUSG00000021745 | Ptprg | -0.4994244 | 0.00171034 |
| ENSMUSG00000061353 | Cxcl12 | -0.4352381 | 0.00185572 |
| ENSMUSG00000104713 | Gbp6 | 0.84638306 | 0.00219007 |
| ENSMUSG00000069874 | Irgm2 | 0.71626571 | 0.00237717 |
| ENSMUSG00000082292 | Gm12250 | 1.53268379 | 0.00413083 |
| ENSMUSG00000024610 | Cd74 | 0.27606469 | 0.00551734 |
| ENSMUSG00000079363 | Gbp4 | 1.02149244 | 0.00551734 |
| ENSMUSG00000073421 | H2-Ab1 | 0.34700732 | 0.00746343 |
| ENSMUSG00000001300 | Efnb2 | -0.3552101 | 0.00766853 |
| ENSMUSG00000028270 | Gbp2 | 0.56673326 | 0.00766853 |
| ENSMUSG00000046879 | Irgm1 | 0.58345349 | 0.00766853 |
| ENSMUSG00000078920 | Ifi47 | 0.69925857 | 0.00766853 |
| ENSMUSG00000094800 | Gm9780 | -1.5599618 | 0.00766853 |
| ENSMUSG00000058756 | Thra | 0.23568945 | 0.01120965 |
| ENSMUSG00000074151 | Nlrc5 | 0.68149172 | 0.01165406 |
| ENSMUSG00000034663 | Bmp2k | -0.2840243 | 0.01233606 |
| ENSMUSG00000044337 | Ackr3 | 0.70235491 | 0.01307198 |
| ENSMUSG00000076431 | Sox4 | 0.3995303 | 0.0130995 |
| ENSMUSG00000078853 | Igtp | 0.94609194 | 0.01585862 |
| ENSMUSG00000105504 | Gbp5 | 0.77012753 | 0.01600789 |
| ENSMUSG00000025279 | Dnase1l3 | 1.50939494 | 0.01602951 |
| ENSMUSG00000072674 | Plac9b | -1.3953635 | 0.02064246 |
| ENSMUSG00000068735 | Trp53i11 | 0.5396164 | 0.02266401 |
| ENSMUSG00000036594 | H2-Aa | 0.32005918 | 0.02565645 |
| ENSMUSG00000032271 | Nnmt | -1.2439187 | 0.03092778 |
| ENSMUSG00000073411 | H2-D1 | 0.20288266 | 0.03092778 |
| ENSMUSG00000023064 | Sncg | -1.3938943 | 0.03168856 |
| ENSMUSG00000041078 | Grid1 | 3.09883815 | 0.03168856 |
| ENSMUSG00000024713 | Pcsk5 | -0.4062393 | 0.03953269 |
| ENSMUSG00000021770 | Samd8 | -0.4109478 | 0.0427535 |
| ENSMUSG00000025278 | Flnb | -0.2587066 | 0.0427535 |
| ENSMUSG00000030787 | Lyve1 | 0.69736487 | 0.04814394 |
| ENSMUSG00000034227 | Foxj1 | -0.5115098 | 0.04814394 |
| ENSMUSG00000054072 | Iigp1 | 0.74988917 | 0.05788149 |
| ENSMUSG00000032796 | Lama1 | 1.33235868 | 0.05978965 |

**Supplementary Table 3. Details of median (RNA-seq TPM) and standard deviation (S.D.) for genes examined in the RNA-seq analysis of PAH patient’s vs healthy controls.**

| **Gene** |  | **Healthy Control** | **PAH** |
| --- | --- | --- | --- |
| **ANXA8** | Median  S.D. | 0.1706  0.1228 | 0.1782  0.1332 |
| **COLQ** | Median  S.D. | 1.163  0.9832 | 0.9547  0.8618 |
| **DNAH1** | Median  S.D. | 2.453  1.310 | 2.186  0.9586 |
| **ITGA6** | Median  S.D. | 42.31  14.10 | 34.31  11.90 |
| **SYT15** | Mean  S.D. | 1.910  0.9632 | 2.005  0.8400 |

**Supplementary Table 4. Selected relative gene expression changes in all genetic or treatment models.**

| **Gene** | ***Bmp9* KO** | ***Bmp9* KO plus BMP9** | **WT plus anti-BMP9** | ***Bmp9* KO plus tmx** | ***Bmp9* KO +**  ***Bmp10* cKO** | ***Bmp*10 cKO plus**  **anti-BMP9** |
| --- | --- | --- | --- | --- | --- | --- |
|  | cf. WT | cf. *Bmp9* KO | cf. IgG | cf. WT plus tmx | cf. WT plus tmx | cf. IgG |
| *Acta2* | - | - | - | - | **DOWN** | na |
| *Des* | - | - | - | **DOWN** | **DOWN** | na |
| *Myh11* | - | - | - | **DOWN** | **DOWN** | na |
| *Anxa8* | **DOWN** | - | - | **DOWN** | **DOWN** | - |
| *Colq* | **DOWN** | - | - | **DOWN** | **DOWN** | **DOWN** |
| *Dnah1* | **UP** | - | - | - | - | - |
| *Itga6* | **UP** | **DOWN** | - | - | **UP** | **UP** |
| *Rbp3* | **UP** | **DOWN** | na | **UP** | **UP** | na |
| *Syt15* | **DOWN** | - | - | **DOWN** | - | **UP** |
| *Tgtp1* | **UP** | **DOWN** | - | - | **UP** | **UP** |
| *Smad6* | - | **UP** | **DOWN** | **DOWN** | **DOWN** | **DOWN** |
| *Adm* | - | **DOWN** | **UP** | - | - | **DOWN** |
| *Edn1* | - | - | **DOWN** | **DOWN** | **DOWN** | **DOWN** |
| *Bmpr2* | - | na | na | **DOWN** | **DOWN** | na |
| *Eng* | - | na | na | **DOWN** | **DOWN** | na |

cf. – compared to.

**Supplementary Figure 1. Right ventricular systolic pressure, cardiomegaly, and splenomegaly assessment of *Bmp9* KO mice. BMP9 and BMP10 treatment of pulmonary microvascular endothelial cells.** (**a**) RNA was isolated from livers of wild type (WT; n=9) and *Bmp9* KO (n=9) mice and *Gdf2* expression was normalised against the housekeeping gene *Hprt*. (**b**) WT (n=5) and *Bmp9* KO (n=13) were anaesthetised using isoflurane and right heart catheterisation performed. Right ventricular systolic pressure (RVSP) was measured. (**c**) Heart weight was assessed as a ratio of femur length in WT (n=11) and *Bmp9* KO (n=7) mice. (**d**) Spleen weight was assessed as a ratio of femur length in WT (n=11) and *Bmp9* KO (n=7) mice. (**e** and **f**) Protein was extracted from WT (n=7) and Bmp9 KO (n=7) lungs. (**e**) Immunoblots were probed for αSMA and desmin, followed by α-tubulin or β-actin to ensure equal loading. (**f**) Densitometry of αSMA and desmin protein expression. (**a** and **f**) Unpaired t-test. ***P≤0.001. Error bars represent mean +/-S.E.M.

**Supplementary Figure 2. BMP9 serum levels and gene expression in *Gdf2^-/-^* mice following recombinant BMP9 treatment.** (**a**) Wild type (WT) and *Gdf2^-/-^* male mice were administered daily for 3-weeks with 30 μg/kg recombinant human BMP9 or vehicle control. Mice were bled at the beginning and end of treatment regime to check BMP9 levels in serum from WT (n=11), *Bmp9* KO plus vehicle (n=7) and *Bmp9* KO plus BMP9 (n=8) using a BMP9 specific ELISA. (**b**) Representative images of lung sections from WT, *Bmp9* KO plus vehicle, and *Bmp9* KO plus BMP9 were immunostained with α-smooth muscle actin (αSMA). Vessels labelled with black arrows. Higher magnification vessel labelled with red arrow indicating αSMA staining. Scale bar = 50 μm. (**c**) RNA was isolated from livers of WT (n=9), *Bmp9* KO plus vehicle (n=7) and *Bmp9* KO plus BMP9 (n=8) mice. *Gdf2* gene expression was normalised against the housekeeping gene, *Hprt*. (**d** - **f**) RNA was isolated from lungs of WT (n=11), *Bmp9* KO plus vehicle (n=6/7) and *Bmp9* KO plus BMP9 (n=6/8) mice. Gene expression was normalised against the housekeeping gene, *Hprt* (**d**) *Acta2*, *Des* and *Myh11* expression. (**e**) *Smad6* expression. (**f**) *Adm* and *Edn1* expression. (**a**, **e,** and **f**) One-way ANOVA. *P≤0.05. Error bars represent mean +/-S.E.M.

**Supplementary Figure 3. BMP9 and BMP10 treatment of pulmonary artery smooth muscle cells and pulmonary microvascular endothelial cells.** (**a** - **d**) Human pulmonary artery smooth muscle cells (PASMCs; n=4 biological replicates) were serum-starved (0.1%) overnight prior to treatment with BMP2 (0.1, 1, 10 ng/ml), BMP9 (0.03, 0.3, 3, 30 ng/ml) and BMP10 (0.03, 0.3, 3, 30 ng/ml) for 8 hours. Gene expression of *ID1* (**a**), *ID2* (**b**) *ITGA6* (**c**) and *SYT15* (**d**) was measured using qPCR, normalised to 3 housekeeping genes (*BACT*, *B2M* and *HPRT*). (**e** and **f**) Human pulmonary microvascular cells (PVMECs; n=4 biological replicates) were serum-starved (0.1%) overnight prior to treatment with BMP9 (0.1, 0.3, 1 ng/ml) and BMP10 (0.1, 0.3, 1 ng/ml) for 8 hours. Gene expression of *ID1* (**e**) and *ID2* (**f**) was measured using qPCR, normalised to 2 housekeeping genes (*B2M* and *HPRT*). (**a**, **b**, **e,** and **f**) One-way ANOVA. *P≤0.05, **P≤0.01, ***P≤0.001, ****P≤0.0001. Error bars represent mean +/-S.E.M.

**Supplementary Figure 4. *ITGA6* and *COLQ* Cox proportional hazard models including age at diagnosis and sex. Cox proportional hazard models** generated with scaled ITGA6 or COLQ including age at diagnosis and sex as co-variates.

**Supplementary Figure 5. No changes in muscularisation following anti-BMP9 treatment.** (**a**) Wild type (WT) mice were administered weekly for 3-weeks with 5 mg/kg BMP9 antibody (anti-BMP9) or equivalent volume of mouse IgG2B (IgG) isotype as a control group. Relevant tissue was collected after 3-weeks. Lung sections were immunostained with α-smooth muscle actin (αSMA). Representative images of lung sections from WT mice administered with IgG or anti-BMP9*.* Vessels labelled with black arrows. Higher magnification vessel labelled with red arrow indicating αSMA staining. Scale bar = 50 μm.

**Supplementary Figure 6. BMP9 and BMP10 levels in *Gdf2^-/-^* and double knockout mice treated with tamoxifen.** (**a** and **b**) *Bmp10^fl/fl^* (WT), *Bmp10^fl/fl^*x*Gdf2^-/-^* (*Bmp9* KO), *Bmp10*^fl/fl^xRosa26^Cre-ERT^ (*Bmp10* cKO) and *Bmp10*^fl/fl^xRosa26^Cre-ERT^x*Gdf2^-/-^* (dKO) mice were treated with tamoxifen once a day for five days with a two-day recovery period followed by a further 5 days at a dose of 40 mg/kg. As a vehicle control, WT mice were administered corn oil for the same period. Mice then underwent right heart catheterisation on day 56. Mice were also bled at day -3, 21 and 56 to assess BMP9 levels. Right atrium was also taken at day 56 to generate BMP10 conditioned media (RACM). Relevant tissue was collected on day 56. (**a**) Serum from WT (corn oil; n=12), WT (tamoxifen; n=19) and *Bmp10* cKO (tamoxifen; n=12) mice bled at day -3, 21 and 56 were assayed for BMP9 levels using a BMP9 specific ELISA. (**b**) Growth factor domain (GFD) BMP10 expression was measured by ELISA. Specificity was assessed by measuring recombinant BMP10 GFD, prodomain BMP10 (proBMP10) and a non-cleavable proBMP10 variant (R313A). (**c**) Conditioned media from right atria collected at day 56 from WT (corn oil; n=12), WT (tamoxifen; n=18), *Bmp9* KO (tamoxifen; n=20), *Bmp10* cKO (tamoxifen; n=14) and dKO (tamoxifen; n=13) was assayed for BMP10 levels using a BMP10 growth factor domain (GFD) specific ELISA. (**d** - **f**) RNA was isolated on day 56 from lungs of WT (corn oil; n=8), WT (tamoxifen; n=8), *Bmp9* KO (tamoxifen; n=8), *Bmp10*-cKO (tamoxifen; n=8) and dKO (tamoxifen; n=8). Gene expression was normalised against the housekeeping gene, *Hprt*. (**d**) *Dnah1* expression. (**e**) *Edn* expression. (**f**) *Adm* expression. (**g**) RNA was isolated from WT (n=4) and *Bmp9* KO (n=6) mice lungs. *Bmpr2*, *Eng* and *Smad6* gene expression was normalised against *Hprt*. (**c**) One-way ANOVA. ****P≤0.0001. Error bars represent mean +/-S.E.M.

**Supplementary Figure 7. Muscularisation in *Gdf2^-/-^* and double knockout mice treated with tamoxifen.** *Bmp10^fl/fl^* (WT), *Bmp10^fl/fl^*x*Gdf2^-/-^* (*Bmp9* KO), *Bmp10*^fl/fl^xRosa26^Cre-ERT^ (*Bmp10* cKO) and *Bmp10*^fl/fl^xRosa26^Cre-ERT^x*Gdf2^-/-^* (dKO) mice were treated with tamoxifen once a day for five days with a two-day recovery period followed by a further 5 days at a dose of 40 mg/kg. As a vehicle control, WT mice were administered corn oil for the same period. Mice then underwent right heart catheterisation on day 56. Lung sections from WT (corn oil), WT (tamoxifen), *Bmp9* KO (tamoxifen), *Bmp10*-cKO (tamoxifen) and dKO (tamoxifen) mice were immunostained with α-smooth muscle actin (αSMA). Vessels labelled with black arrows. Higher magnification vessel labelled with red arrow indicating αSMA staining. Scale bar = 50 μm.

**Supplementary Figure 8. Conditional knockout mice treated with anti-BMP9 don’t have multiple organ anomalies or reduced smooth muscle muscularisation.** (**a** - **f**) *Bmp10*^fl/fl^xRosa26^Cre-ERT^ (*Bmp10* cKO) were treated with tamoxifen once a day for five days with a two-day recovery period followed by a further 5 days at a dose of 40 mg/kg. On day 21 mice were dosed weekly for 2-weeks with 5 mg/kg BMP9 antibody (anti-BMP9) or equivalent volume of mouse IgG2B (IgG) isotype as a control. Mice then underwent right heart catheterisation on day 42. Relevant tissue was collected on day 42. (**a**) Heart weight was assessed as a ratio of femur length in IgG (n=9) and anti-BMP9 (n=9) treated mice. (**b**) Spleen weight was assessed as a ratio of femur length in IgG (n=9) and anti-BMP9 (n=9) treated mice. (**c**) Heart rate was measured in IgG (n=9) and anti-BMP9 (tamoxifen; n=8) treated mice. (**d**) Measurement of cardiac output in IgG (n=8) and anti-BMP9 (tamoxifen; n=8) treated mice. (**e**) Right ventricular systolic pressure (RVSP) was measured in IgG (n=9) and anti-BMP9 (n=7) treated mice. (**f** and **g**) (**f**) Lung sections from *Bmp10* cKO treated with IgG or anti-BMP9 were immunostained with α-smooth muscle actin (αSMA). Vessels labelled with black arrows. Higher magnification vessel labelled with red arrow indicating αSMA staining. Scale bar = 50 μm. (**g**) Quantification of non-muscularised vessels as a percentage of arteries associated with alveolar ducts in IgG (n=8) and anti-BMP9 (n=9) treated mice. 20 arteries were counted per animal. (**b**) Unpaired t-test. *P≤0.05. Error bars represent mean +/-S.E.M.


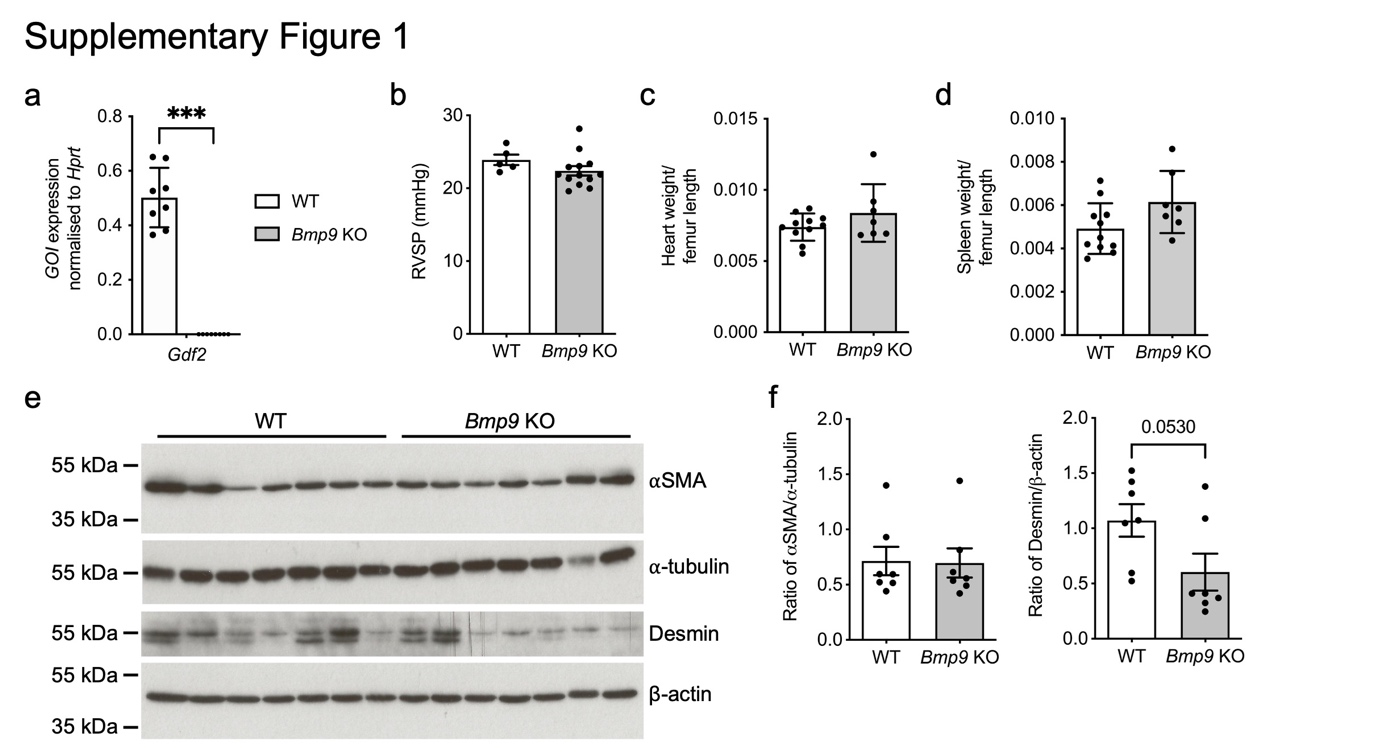


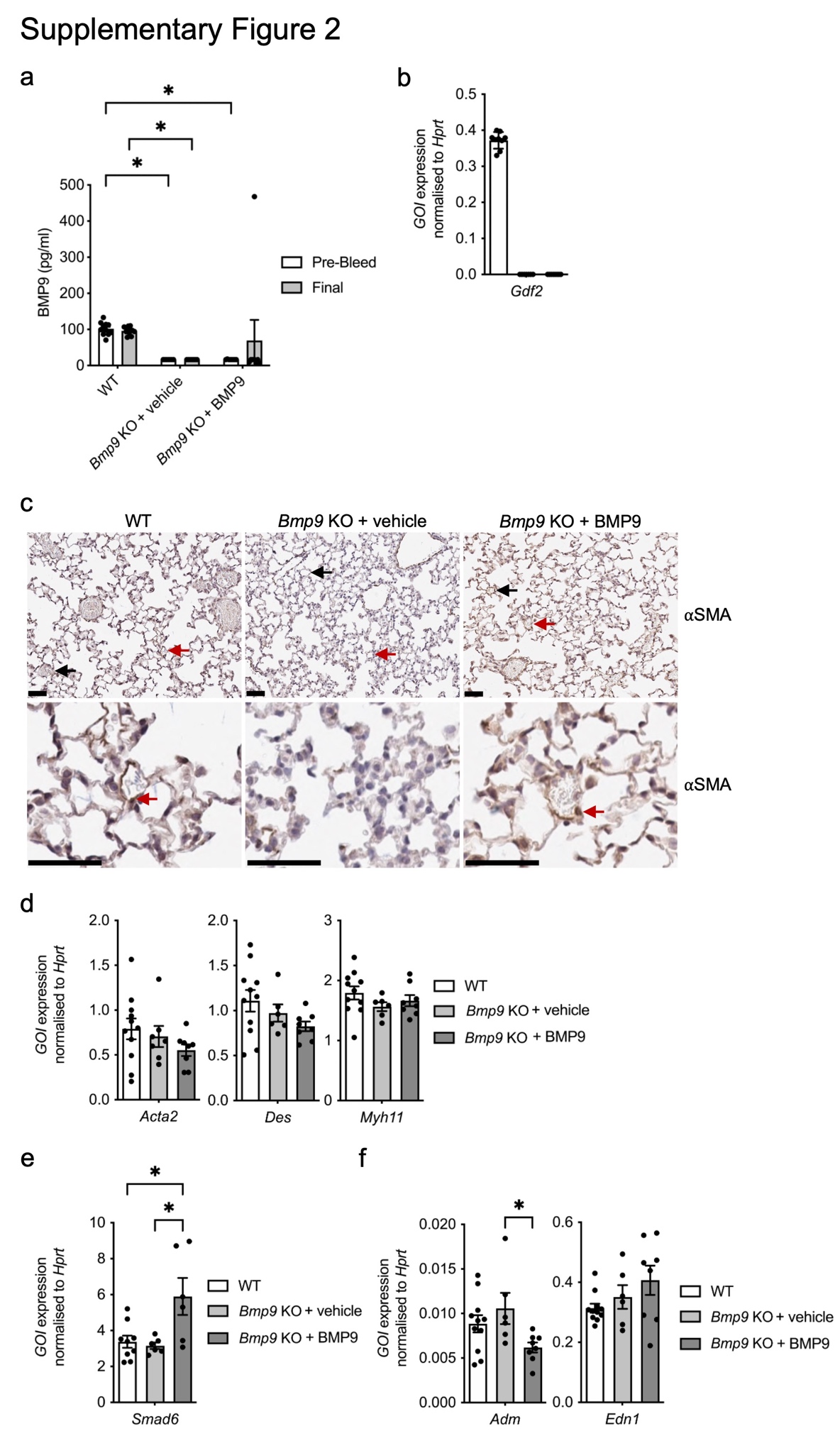


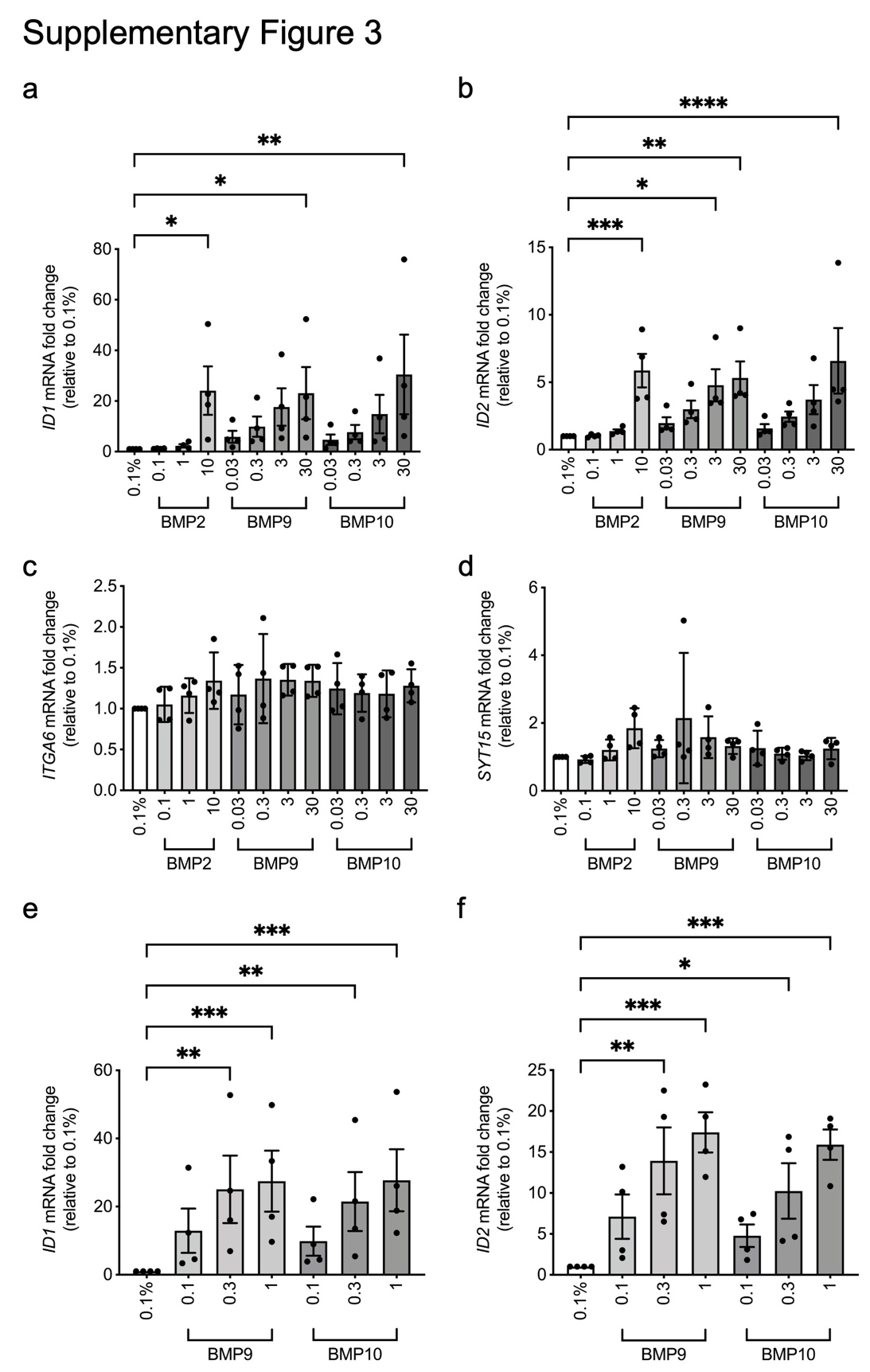


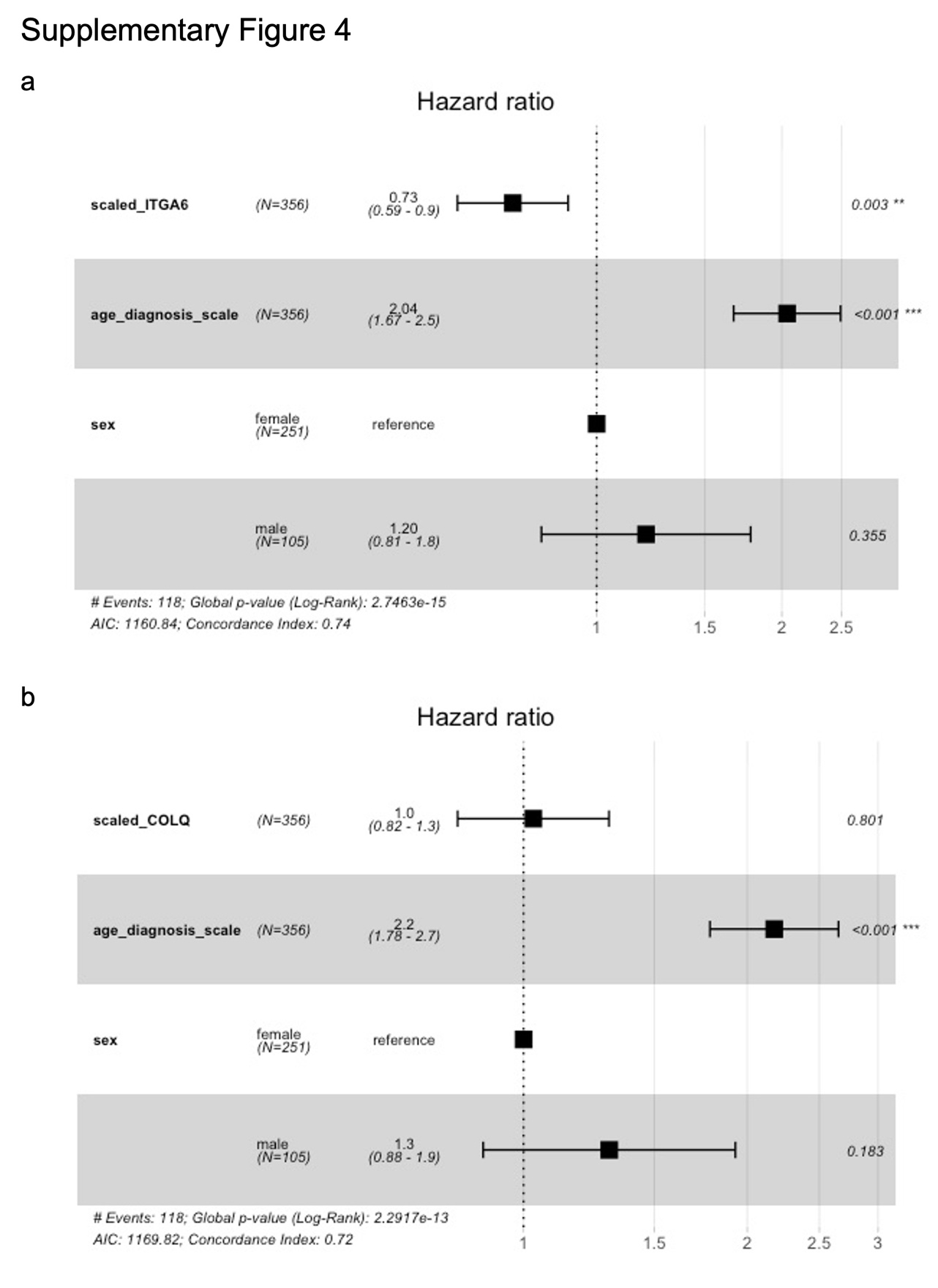


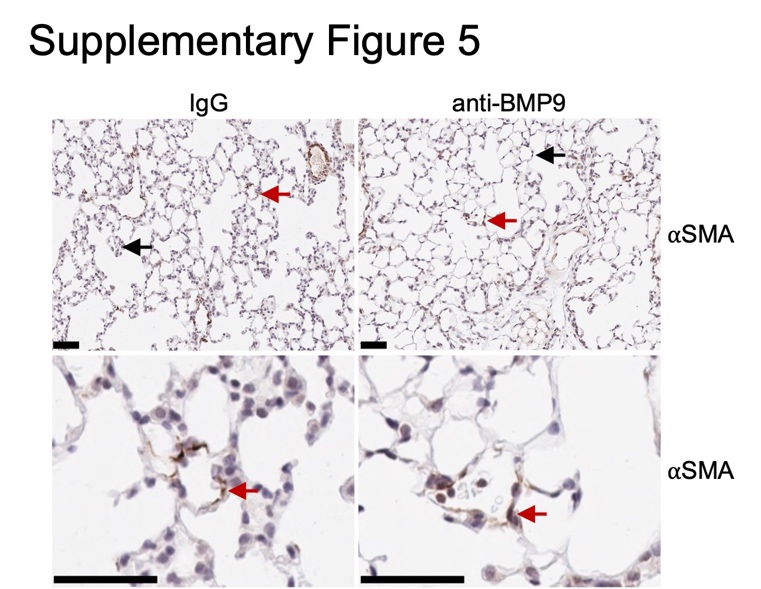


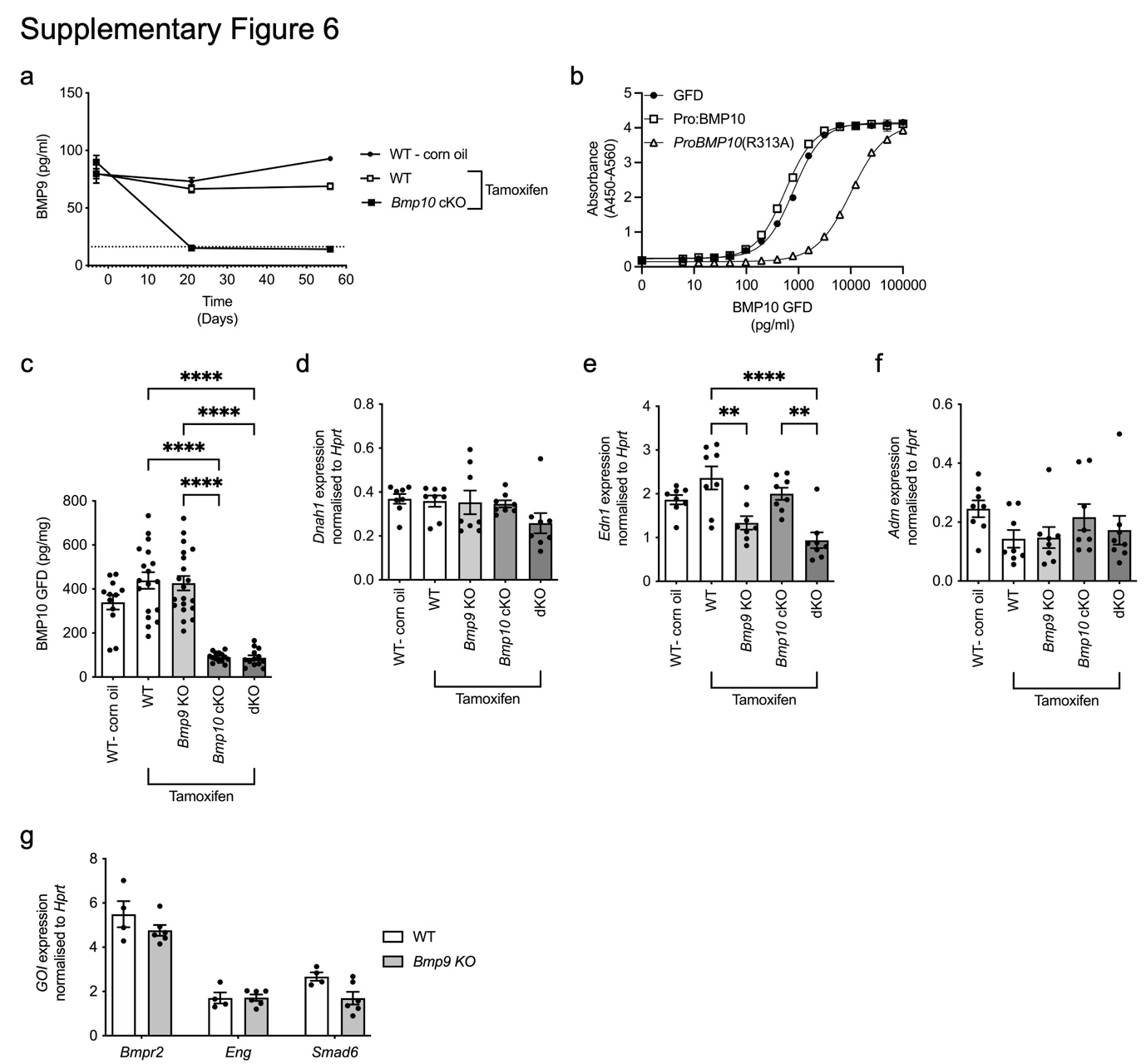


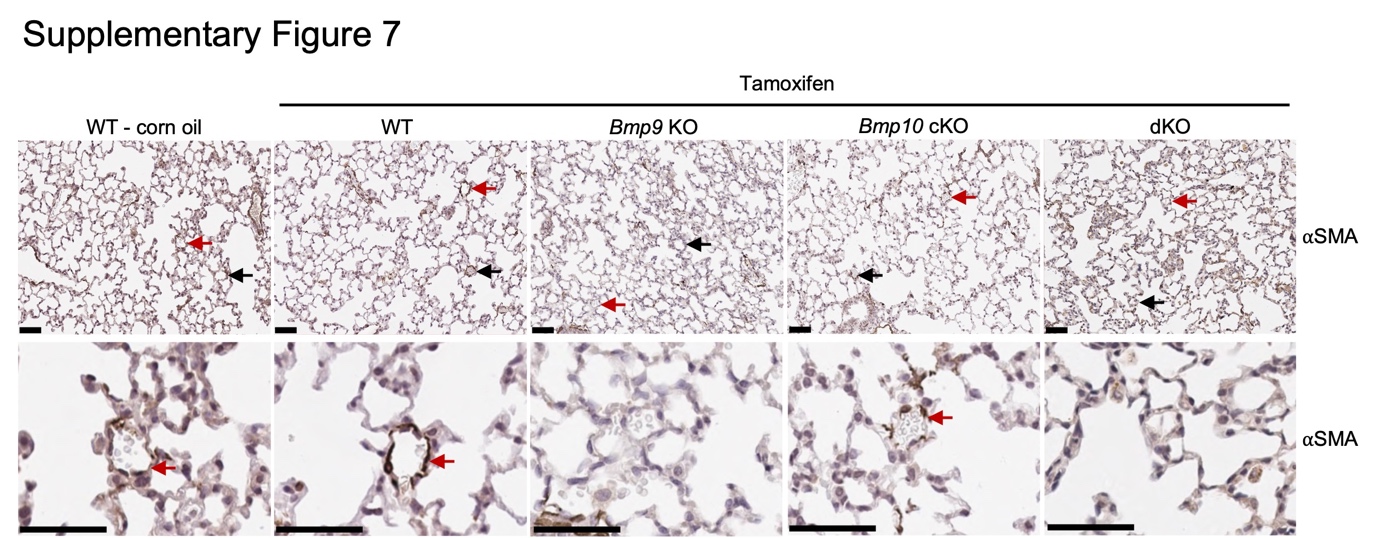


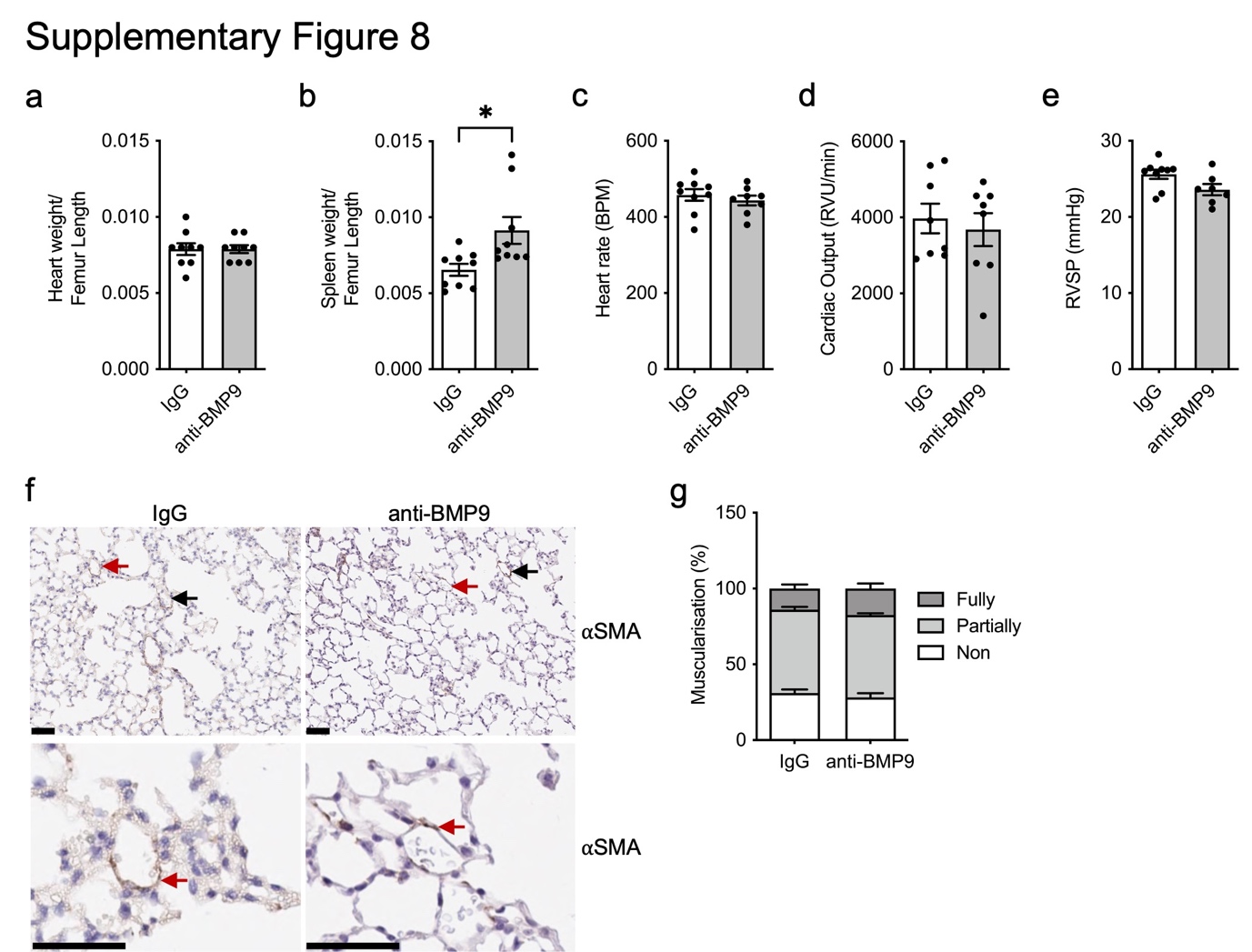

Supplement: Supplementary file 1 — Supplementary Material 1 [file 10456_2025_10017_MOESM1_ESM.docx]
